# Effect of Lung Cancer Screening, Smoking Cessation, and Cessation Smartphone App to Health-Related Quality of Life Among Heavy Smokers: Randomized Controlled Trial

**DOI:** 10.2196/81687

**Published:** 2026-01-20

**Authors:** Antti Kurtti, Sanna Iivanainen, Riitta Kaarteenaho, Heidi Andersen, Antti Jekunen, Tuula Vasankari, Jussi Koivunen

**Affiliations:** 1 Cancer Center, Oulu University Hospital, Kajaanintie 50, Oulu, 90029, Finland, 358 504182666, 358 83156449; 2 Faculty of Medicine, University of Oulu, Oulu, Finland; 3 Medical Research Center Oulu, Oulu, Finland; 4 Research Unit of Biomedicine and Internal Medicine, University of Oulu, Oulu, Finland; 5 Department of Oncology and Radiotherapy, Vaasa Central Hospital, Vaasa, Finland; 6 Faculty of Medicine, University of Turku, Turku, Southwest Finland, Finland; 7 FILHA, Helsinki, Finland

**Keywords:** lung cancer screening, smoking cessation, mobile app, low-dose computed tomography, health-related quality of life

## Abstract

**Background:**

Lung cancer screening with low-dose computed tomography (LDCT) among heavy smokers can decrease lung cancer mortality. Smoking cessation intervention is recommended within the screening program; however, the methods for smoking cessation in the LDCT screening context are not well established. We have previously shown that a novel smartphone app can increase the chance for smoking cessation along with lung cancer screening. The effects of lung cancer screening, smoking cessation, and the use of smartphone apps on health-related quality of life (HRQoL) are widely unknown.

**Objective:**

This study aims to investigate the effect of lung cancer screening, smoking cessation, and the use of smoking cessation app on HRQoL, an exploratory end point of the low-dose computed tomography screening for lung cancer combined to different smoking cessation methods in Finland (LDCT-SC-FI) study.

**Methods:**

This study was conducted as a part of the LDCT-SC-FI (NCT05630950), which was a randomized controlled trial investigating different smoking cessation methods in participants undergoing lung cancer screening with LDCT. The main inclusion criteria included an age of 50‐74 years, a marked smoking history (smoked ≥15 cigarettes per day for ≥25 years or smoked ≥10 cigarettes per day for ≥30 years), an active smoking status, and access to a smartphone. The recruitment was carried out by newspaper and internet advertisements and informing relevant health care units at hospital districts. The study participants (n=200), all at Oulu University Hospital, were randomized in 1:1 fashion to a yearly LDCT with standard smoking cessation (written material) or a stand-alone smartphone app–based cessation. HRQoL, an exploratory study end point, was assessed at baseline and at 1 year with Quality of Life Questionnaire Core 30 (QLQ-C30) and EQ-5D.

**Results:**

In total, 199 and 186 individuals had both questionnaires completed at baseline and at 1 year, respectively. We did not detect a change in HRQoL between the time points using QLQ-C30 global health status score or EQ-5D index score. Smoking cessation at 1-year time did not affect QLQ-C30 global health status or EQ-5D. We observed improved quality of life scores by EQ-5D at 1 year (control: mean 0.720, SD 0.197 vs app: mean 0.799, SD 0.197; improved in 17/93, 18% of controls vs 29/93, 31% in app arm), while there was no difference in means at baseline. Smartphone app arm reported reduced pain (EQ-5D effect size [ES] 0.049, 95% CI 0.006‐0.12; *P*=.01; adjusted ES 0.026; *P*=.007; QLQ-C30 ES 0.076, 95% CI 0.02‐0.16; *P*<.001; adjusted ES 0.05; *P*=.02) and increased mobility (EQ-5D ES 0.031, 95% CI 0.01‐0.09; *P*=.02; adjusted ES 0.037; *P*=.008) at 1 year. The number of completed questionnaires in the app was associated with improved HRQoL by EQ-5D (ES 0.073, 95% CI 0.00‐0.180; *P*=.04; adjusted ES 0.071; *P*=.04).

**Conclusions:**

This is the first study to test a smoking cessation smartphone app in the context of lung cancer screening. The use of the developed app correlated with improved HRQoL, mainly by decreased pain and fatigue. To conclude, the studied app provides a feasible and effective cessation intervention that is readily implementable in population-based lung cancer screening programs, with enhanced health benefits beyond smoking cessation.

## Introduction

The premier cause of cancer mortality in the Western world is lung cancer, of which smoking is the single most important risk factor [1]. Lung cancer is typically diagnosed at an advanced stage, preventing curative intent treatments. Using low-dose computed tomography (LDCT) for lung cancer screening in individuals with substantial smoking history can decrease lung cancer mortality [2,3]. Concordantly, smoking cessation interventions are recommended in lung cancer screening programs and may come with enhanced efficiency, regardless of screening results [4-7]. Approximately 7%‐23% of participants in LDCT programs achieve smoking cessation [8]. Still, optimal smoking cessation methods in the context of lung cancer screening are not well established.

Possible health-related quality of life (HRQoL) effects and losses are important to evaluate in cancer screening trials [9]. There is moderate evidence that, compared with no screening, persons receiving LDCT screening do not have worse general HRQoL or distress over 2 years of follow-up, and anxiety may even be lowered. However, consequences might differ based on screening results, at least in the short term [10-12]. Observed negative psychological effects diminished over time, and there were no known negative long-term effects on HRQoL [13,14].

Opportunities for using digital tools to access smoking cessation treatment are evolving rapidly due to the expansion in the proportion of the global population with access to a mobile phone. Data imply that apps that provide personalized and adaptive as well as interactive support may be more effective in promoting engagement [15-17]. Smartphone apps have reached abstinence odds ratios (ORs) of 1.25‐1.51 (95% CI 0.99‐1.56, 1.24‐1.84) in meta-analyses [18]. Their efficacy can be enhanced with pharmacotherapy and physical participant recruitment [19]. We have recently shown that a novel smartphone app can increase smoking cessation with an OR of ~3 at 3 and 6 months when applied within LDCT screening for lung cancer [20].

Several studies have indicated that smoking is related to lower HRQoL and mental well-being [21-23]. However, studies on the impact of smoking cessation interventions on HRQoL have provided mixed results, some concluding that it may improve, yet others showing negative or no changes [24-26]. Smoking cessation may have a positive effect on physical and general health but no significant effects on mental aspects [24]. Nevertheless, it might take years of abstinence for smokers’ HRQoL to be equal to nonsmokers’ [24,27].

Low-dose computed tomography screening for lung cancer combined with different smoking cessation methods in Finland (LDCT-SC-FI) is a randomized controlled trial investigating smoking cessation with a smartphone app compared to written cessation materials in individuals participating in LDCT screening for lung cancer. The core concepts behind the developed app include cognitive behavioral (enhancing self-awareness, problem-solving skills, goal setting, and coping with cravings) and social cognitive theories and acceptance and commitment therapy as well as mindfulness. Therefore, the effects of smartphone apps could extend beyond smoking cessation.

This study aims to investigate changes in HRQoL, an exploratory end point of the LDCT-SCI-FI trial. We investigated the changes in HRQoL using 3 different patient-reported outcome measures (PROMs). Since the LDCT-SC-FI study focused on 2 main themes (feasibility of LDCT screening and smoking cessation with a smartphone app), the results were analyzed to ascertain what effects participation in screening, smoking cessation, and use of the interventional app might have on HRQoL outcomes.

## Methods

### Study Design

This study was conducted as a part of the LDCT-SC-FI (NCT05630950), which is a randomized controlled trial investigating different smoking cessation methods in participants undergoing lung cancer screening with LDCT. The study participants were randomized in a 1:1 fashion to a yearly LDCT with standard smoking cessation (written material) or the same LDCT screening approach with a smartphone app–based smoking cessation (experimental). The study was powered (80%) with 156 participants to detect a 15% difference with α of .1 (75% vs 90%) in the number of active smokers at 3 and 6 months after inclusion using an online calculator. With the expected dropout rate, the sample size was adjusted to 200.

### Ethical Considerations

The study was approved by the ethics committee of Northern Ostrobothnia Hospital District (EETTKM 21/2022) and was prospectively registered at ClinicalTrials.gov (NCT05630950). All the participants signed an informed consent before any study procedures. The study participants were not compensated for their participation. Of note, LDCT lung cancer screening is not among the publicly funded cancer screenings in Finland. The study was conducted in accordance with the Declaration of Helsinki [28] and Good Clinical Practice guidelines [29]. Participant data were deidentified prior to analysis. Unique study codes were assigned to each participant, while identifying information was removed. All results are reported in aggregate form to protect participant confidentiality. Consent for publication has been granted by identifiable individuals.

### Participants

Eligibility followed closely to the Nederlands-Leuvens Longkanker Screenings Onderzoek (NELSON) lung cancer screening trial criteria [2]. The main inclusion criteria included an age of 50‐74 years, a marked smoking history (smoked ≥15 cigarettes per day for ≥25 years or smoked ≥10 cigarettes per day for ≥30 years), an active smoking status (smoking during the last 2 weeks including regular [daily smoking] and occasional [nondaily smoking] habits), and access to a smartphone (iPhone or Android). The main exclusion criteria, as in the NELSON trial, included a moderate or bad self-reported health; current or past melanoma, lung, renal, or breast cancer; and a chest computed tomography (CT) examination within 1 year.

### Recruitment and Randomization

All the participants were recruited at the Oulu University Hospital (from November 18, 2022, to April 14, 2023; the final study visit occurred on March 20, 2024). The recruitment was carried out by newspaper and internet advertisements and informing relevant health care units at the hospital district. A physical screening visit was performed at the site where participating individuals signed the informed consent. Eligibility was verified by a study nurse according to a checklist. Study participants did not receive any compensation for participation, and all the study procedures were free of charge. Eligible participants were randomized by study nurses with block method (sequentially numbered containers with a block size of 10, study arm written on a paper in an opaque, sealed envelope) in 1:1 fashion with stratification according to pack years (<30 or ≥30 pack years) and age (<65 or ≥65 years) to smartphone-based smoking cessation and control (written smoking cessation material) arms. The stratification factors were selected based on the assumption that bias could be generated by (1) adoption of smartphone use in older people and (2) higher pack years to be associated with a lesser likelihood of smoking cessation. The random allocation sequence (randomization envelopes and numbered blocks) was generated by the investigators (JK and SI) to ensure concealment. Because of an inability to blind the study participants from the intervention, as well as self-reported smoking cessation being the primary end point of the study, the study personnel were not blinded.

### Outcomes

Data collected at baseline included standard demographics and detailed smoking history. Self-reported smoking cessation status was verified by phone at 3 and 6 months, and as a part of physical visit at 1 year. Another physical visit and LDCT screening investigation took place at 1 year. Quality of life (QoL) questionnaires were collected after randomization at baseline and at 1 year.

The primary outcomes of the study were self-reported smoking cessation at 3 and 6 months (±1 month), which has been recently reported, and study details are available in this publication [20]. Furthermore, secondary outcomes of the study included efficiency of different smoking cessation methods in the reduction of smoking, sensitivity and positive predictive value of CT screening, and costs related to CT screening, which are reported in previous publications. HRQoL assessed with different PROMs was an exploratory end point of the study and was not specified in detail in the protocol. Since the study focused on 2 main themes (smoking cessation with a smartphone app and feasibility of LDCT lung cancer screening), we planned to investigate HRQoL in the context of smoking cessation, study arm, and temporally. All the analysis was carried out according to specific instructions by the HRQoL questionnaire. ED-5D index scores and QLQ-C30 global health status (GHS) have standardized population-based cutoffs (5% for EQ-5D and 10% for the QLQ-C30), which were used in the main analysis of HRQoL. If any of the main analyses showed statistically significant differences temporally, or by study arm, or smoking status at 1 year, detailed analysis of specific subscales or scores would be carried out.

### Procedures

The developed smoking cessation app called Suunta supports smokers in the cessation process and aids them to retain a smoking-free lifestyle (Multimedia Appendix 1). The theoretical and functional concept was created by the study team members, and the technical execution was done under subcontract by a company specialized in mobile app development (Techinspire). The stand-alone app is Android- and iPhone-compatible with cloud-based database back-up. The Suunta app was downloaded on participants’ smartphones on the randomization visit, which was assisted by the study nurse. The Suunta smartphone app was offered for the individuals in the control arm after 6 months of follow-up.

The written materials used for smoking cessation are based on the Finnish Current Care Guideline for Prevention and Treatment of Smoking and Nicotine Addiction. A printed version of the patient guide was handed out to all the study participants. At the 6-month smoking status call, participants in the control arm were offered the possibility to start using the smoking cessation app. No other counseling for smoking cessation was provided to the participants during the study visits regardless of the study arm.

The LDCT-SC-FI study protocol for LDCT (Multimedia Appendix 2) follows the NELSON study protocol [2]. In brief, all the study participants undergo LDCT screening within 6 weeks from the randomization, and the next LDCT is scheduled for 1 year.

### HRQoL Measurements

Study participants were assessed for HRQoL with European Organisation for Research and Treatment of Cancer (EORTC) Quality of Life Questionnaire Core 30 (QLQ-C30)+Quality of Life Questionnaire Lung Cancer 13 (QLQ-LC13) and EuroQol EQ-5D-3L at baseline and at 1 year. The participants completed the questionnaires at a physical visit using paper format. HRQoL and changes therein were collected as the QLQ-C30 GHS and EQ-5D index scores at baseline and 1 year in all participants as well as according to smoking status at 1 year and study arms. HRQoL-related secondary outcomes included the means and changes in the QLQ-C30 functional scales as well as in the specific symptom scores of QLQ-C30, QLQ-LC13, and EQ-5D. These were carried out only if the overall score was found to be significant at a 1-year time point.

The EORTC QLQ-C30 (version 3.0) and QLQ-LC13 questionnaires were scored according to official EORTC scoring manuals. In brief, all the scales and single-item measures undergo linear transformation to range from 0 to 100, so that a high score for the functional and GHS scale represents a high level of functioning or QoL, while the score for the symptom scales and single items represents a high level of symptomatology or problems. A 10-point change in scores is considered meaningful, so a 10% cutoff was selected for analysis [30].

With EQ-5D-3L questionnaires, single scores were reported by 3-grade answers representing no to severe problems. For the EQ-5D index score, all the scores were transformed to a single raw score between 0 and 1 according to the official scoring manual. Raw scores were transformed to a country-specific index score (Finland) using a Microsoft Excel-based calculator. A 5% change in EQ-5D index score is considered significant in Finland, and this was selected as a primary measure [31,32].

The use of the app and its association with HRQoL changes observed in the QLQ-C30 GHS and EQ-5D index scores were studied in the experimental arm. The smartphone app included weekly symptom questionnaires, and the frequency of app use was investigated by analyzing the number of completed symptom questionnaires by 24 weeks.

### Statistical Analysis

Data analysis was carried out using SPSS (version 29.0.1; IBM Corp). All the statistical analyses were carried out blinded to the group allocation. Reliability of HRQoL data was evaluated with Cronbach α, with values of <0.6 considered poor or unacceptable. Paired 2-tailed *t* test was used to compare means of 2 related groups (change over time) and estimate *P* value and effect size (ES) with 95% CIs. For categorized variables, Pearson chi-square test was used to estimate *P* values, and 1-way ANOVA for ES with 95% CIs. For continuous variables, 1-way ANOVA was applied to estimate the means, *P* values, and ES with 95% CIs. One-way ANOVA was also applied in the analysis of variables with more than 2 categories to estimate *P* values and ES with 95% CIs. Adjusted analysis for *P* values and ES was carried out using analysis of covariance corrected for relevant baseline factors. Since the statistical software used does not provide CIs for adjusted analysis of covariance, these are not provided. Ordinal regression analysis with the proportional odds model was used to estimate ORs, 95% CIs, and *P* values. The generated model was evaluated with model fitting, goodness-of-fit, and test-of-parallel-lines test. *P* values of <.05 were considered statistically significant. For the ES, partial η^2^ (small: 0.01; medium: 0.06; large: 0.14) or Cohen *d* (small: 0.2; medium: 0.5; large: 0.8) were used to estimate the magnitude of the effect. Missing data were not replaced.

## Results

### Demographics

**Figure 1.**
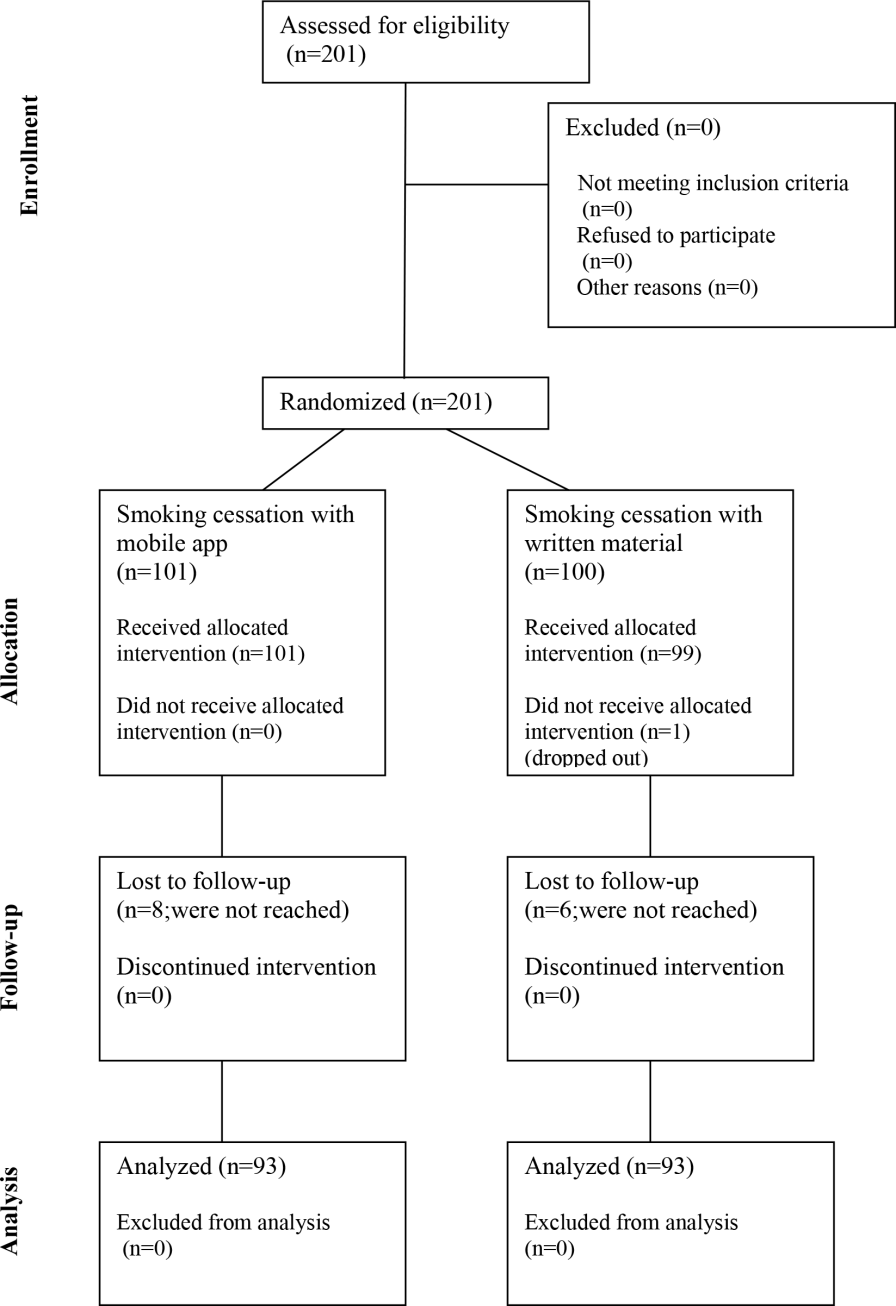
Study flowchart of the LDCT-SC-FI, which was a randomized controlled trial investigating different smoking cessation methods in participants undergoing lung cancer screening with low-dose computed tomography. LDCT-SC-FI: low-dose computed tomography screening for lung cancer combined with different smoking cessation methods in Finland.

The recruitment was initiated on November 18, 2022, the last participant was included on April 14, 2023, and the final study visit occurred on March 20, 2024. The median age was 60 (IQR 56-66) years, and 51% (102/201) of them were female. In smoking-related demographics, the median number of pack years was 31 (IQR 24-40), and the number of smoked cigarettes per day was 15. The detailed demographics are presented in Table S1 in Multimedia Appendix 3 and the study flowchart in Figure 1.

### Temporal Changes in HRQoL During Screening

All the study participants (n=200) underwent LDCT screening at baseline and 183 of the eligible 196 (93%) at 1 year. At baseline, all the study participants (n=200) had 3 QoL (EQ-5D and QLQ-CO30+LC13) questionnaires filled, and all (n=186) came to the 1-year study visit (Figure 1). There were 14 dropouts for the 1-year visit (experimental: n=8; control: n=6), 4 by protocol-defined exclusion and 10 by self-willed withdrawal. The overall HRQoL scores and their changes between baseline and 1 year were analyzed. Data reliability was considered good or acceptable for overall HRQoL scores (Table S2 in Multimedia Appendix 3). Among all study participants, there was no statistically significant difference between the means of either score at baseline or at 1 year. However, there was a slight decrease in GHS over time. Most participants experienced no changes in either of their scores (GHS: 120/186, 65%, EQ-5D index score: 90/186, 48%). The subgroup of participants experiencing either improved or decreased HRQOL scores was quite balanced (Table 1). Furthermore, the association of EQ-5D index score change (5%) and GHS change (10%) was evaluated, and they showed a good correlation (Table S3 in Multimedia Appendix 3).

**Table 1.** Study participants were assessed for HRQoL^a^ with European Organisation for Research and Treatment of Cancer QLQ-C30^b^ and EuroQol EQ-5D-3L at baseline and at 1 year at a physical visit^c^.

	Baseline	At 1 year	Effect size^d^ (95% CI)	*P* value^e^
QLQ-C30 GHS^f^, mean (SD)	73.21 (15.83)	71.64 (19.71)	0.09 (−0.6 to 0.23)	.23
QLQ-C30 GHS (10% change), n (%)	—^g^	186 (100)	—	—
Improved		33 (18)		
No change		120 (65)		
Decrease		33 (18)		
EQ-5D index score, mean (SD)	0.763 (0.195)	0.760 (0.201)	0.02 (−0.13 to 0.16)	.80
EQ-5D index score (5% change), n (%)	—	186 (100)	—	—
Improved		46 (25)		
No change		90 (48)		
Decrease		50 (27)		

a HRQoL: health-related quality of life.

b QLQ-C30: Quality of Life Questionnaire Core 30.

c HRQoL and changes therein were collected as the QLQ-C30 GHS and EQ-5D index scores. A 10-point change in scores of QLQ-C30 is considered meaningful; thus, a 10% cutoff was selected, while a 5% change in EQ-5D index score is considered significant in Finland.

d Cohen *d*.

e Paired samples *t* test.

f GHS: global health status.

g Not applicable.

### HRQoL According to Smoking Status

Since smoking cessation was the primary end point of the study, we wanted to analyze whether smoking cessation would have an impact on HRQoL outcomes. Smoking status was available for all study participants (n=186) coming to the study visit at 1 year (Figure 1). We assessed the association of smoking cessation to GHS and EQ-5D scores. Smoking cessation status showed no statistically significant difference in either of the HRQoL measures at baseline or at 1 year. Furthermore, we detected no difference according to smoking status in participants whose scores improved, decreased, or remained the same (Table 2).

**Table 2.** Changes in quality of life based on QLQ-C30^a^ and EQ-5D between baseline and at 1 year according to smoking status (at 1 year) irrespective of study arm.

	Smoker	Nonsmoker	Effect size (95% CI)^b^	*P* value^c^
QLQ-C30 GHS^d^, mean (SD)				
Baseline	72.78 (15.97)	74.14 (16.72)	0.001 (0.00-0.003)	.70
1 year	71.44 (19.48)	72.70 (21.24)	0.001 (0.00-0.03)	.75
Mean change from baseline	−1.592 (17.09)	−1.437 (22.28)	0.00 (0.00-0.003)	.97
QLQ-C30 GHS (10% change), n (%)	157 (100)	29 (100)	0.006 (0.00-0.05)	.57
Improved	26 (17)	7 (24)		
No change	102 (65)	18 (62.1)		
Declined	29 (19)	4 (14)		
EQ-5D index score, mean (SD)				
Baseline	0.759 (0.196)	0.767 (0.204)	0.00 (0.00-0.02)	.86
1 year	0.760 (0.201)	0.758 (0.202)	0.00 (0.00-0.005)	.96
Mean change from baseline	−0.002 (0.17)	−0.009 (0.17)	0.00 (0.00-0.02)	.85
EQ-5D index score (5% change), n (%)	157 (100)	29 (100)	0.002 (0.00-0.04)	.80
Improved	40 (26)	6 (21)		
No change	76 (48)	14 (48)		
Declined	41 (26)	9 (31)		

a QLQ-C30: Quality of Life Questionnaire Core 30.

b Partial eta-square.

c ANOVA or Pearson chi-square test.

d GHS: global health status.

### HRQoL According to Randomization Arm

Next, we assessed HRQoL outcomes according to study arm. There was no difference either in the GHS or EQ-5D index score at baseline. At 1 year, we observed a difference between the study arms in the means of both the GHS (68.46 vs 74.82; partial η^2^=0.026, 95% CI 0.00‐0.09; *P*=.03) and the EQ-5D index scores (0.720 vs 0.799; partial η^2^=0.039, 95% CI 0.003‐0.11; *P*=.007), with individuals randomized to the app arm showing higher scores. However, the statistical significance was lost for the GHS after adjusting for baseline scores. Using the 3-class classification of scores (improved, unchanged, or declined), there was a statistically significant difference between the study arms in the portion of participants whose scores improved and declined by EQ-5D (17/186, 18% vs 29/186, 31%; 31/186, 33% vs 19/186, 20%; partial η^2^=0.032, 95% CI 0.001‐0.10; *P*=.049). A similar trend was observed with GHS (improved: 11/186, 12% vs 22/186, 24%), but this did not reach statistical significance (Table 3).

**Table 3.** Changes in overall quality of life based on QLQ-C30^a^ and EQ-5D questionnaires between randomization arms^b^.

	Control^c^	App^d^	*P* value^e^	Effect size^f^ (95% CI)	Adjusted *P* value^g^	Adjusted effect size^g^ (95% CI)
QLQ-C30 GHS^h^, mean (SD)						
Baseline	70.29 (15.85)	74.17 (16.31)	.09	0.015 (0.00-0.06)	—^i^	—
1 year	68.46 (19.77)	74.82 (19.23)	.03	0.026 (0.00-0.09)	.18	0.01
Mean change from baseline	−2.330 (16.41)	−0.807 (19.39)	.56	0.002 (0.00-0.03)	—	—
QLQ-C30 GHS (10% change), n (%)	93 (100)	93 (100)			—	—
Improved	11 (12)	22 (24)	.11	0.016 (0.00-0.07)		
No change	64 (69)	56 (60)	—	—		
Declined	18 (19)	15 (16)	—	—		
EQ-5D index score, mean (SD)						
Baseline	0.752 (0.197)	0.759 (0.206)	.82	0.000 (0.00-0.02)	—	—
1 year	0.720 (0.197)	0.799 (0.197)	.007	0.039 (0.003-0.11)	.003	0.047
Mean change from baseline	−0.033 (0.16)	0.027 (0.17)	.01	0.032 (0.001-0.10)	—	—
EQ-5D index score (5% change), n (%)	93 (100)	93 (100)			—	—
Improved	17 (18)	29 (31)	.049	0.032 (0.001-0.10)		
No change	45 (48)	45 (48)	—	—		
Declined	31 (33)	19 (20)	—	—		

a QLQ-C30: Quality of Life Questionnaire Core 30.

b Comparison is carried out based on means at individual time points, changes from baseline, means adjusted to baseline values, and by using clinically significant threshold changes (QLQ-C30 10% change; EQ-5D 5% change).

c Control: smoking cessation with written material.

d App: smartphone app–based smoking cessation.

e ANOVA or Pearson chi-square test.

f Partial eta-square.

g Analysis of covariance test adjusted for baseline QLQ-C30 GHS or EQ-5D index score.

h GHS: global health status.

i Not applicable.

We also carried out an ordinal regression analysis of EQ-5D index change (5%) with baseline factors and smoking status at 1 year. The model performance was poor with high *P* values for model fit, while goodness of fit and test of parallel lines showed both *P* values over >.5. Of the analyzed factors, only randomization arm (OR −0.668, 95% CI −1.227 to −0.109; *P*=.02) significantly predicted EQ-5D change (Table 4).

Since Suunta app was also offered to the control arm after 6 months, we wanted to assess whether this would affect the observed results. The app use was assessed at the 1-year study visit, and 14 participants (15% of the controls) reported having used the app. Removing these individuals from the analysis did not markedly alter the results compared to the intent-to-treat analysis. In brief, EQ-5D mean index scores and QLQ-C30 GHS means were similar at baseline, while there was a statistically significant difference at 1 year. Furthermore, according to 3-class classification of EQ-5D or QLQ-C30, very similar results favoring the app were observed while this was only statistically significant with QLQ-C30 (partial η^2^=0.027, 95% CI 0.00‐0.09; *P*=.09; partial η^2^=0.023, 95% CI 0.00‐0.08; *P*=.04, respectively; Table S4 in Multimedia Appendix 3).

**Table 4.** Ordinal regression analysis for clinically significant EQ-5D index change (5%) at 1 year.

	OR^a^ (95% CI)	*P* value^b^
Age	0.034 (−0.018 to 0.087)	.20
Sex		
Female	−0.001 (−0.633 to 0.630)	.10
Male	0	—^c^
Relationship status		
Single	−0.082 (−0.713 to 0.548)	.83
In a relationship	0	—
ICT^d^ skills		
Novice	−0.962 (−2.099 to 0.174)	.10
Average	−0.124 (−0.700 to 0.452)	.67
Experienced	0	—
How many cigarettes per day	0.004 (−0.072 to 0.080)	.92
Pack years	−0.003 (−0.041 to 0.035)	.88
Fagerström test	−0.23 (−0.337 to 0.291)	.89
Smoking status at 1 year		
Nonsmokers	−0.363 (−1.130 to 0.405)	.35
Smoker	0	—
Randomization arm		
Control	−0.668 (−1.227 to −0.109)	.02
App	0	—

a OR: odds ratio.

b Ordinal regression analysis with proportional odds model; model fitting: *P*=.36; goodness-of-fit: *P*=.38; test of parallel lines: *P*=.06.

c Not applicable.

d ICT: information and communication technology.

Data reliability of QLQ-C30-, LC13-, and EQ-5D–defined functioning or symptom subscales was considered good to questionable, and only a few symptoms (hemoptysis, sore mouth, and alopecia) produced poor or unacceptable scores (Table S2 in Multimedia Appendix 3). In the analysis for the QLQ-C30 functional scales, we observed no difference at baseline. At 1 year, there were statistically significant differences between study arms in role (mean 81.70, SD 25.20 vs mean 89.13, SD 19.83; partial η^2^=0.026, 95% CI 0.00‐0.09; *P*=.03) and social (mean 86.02, SD 19.24 vs mean 93.55, SD 15.74; partial η^2^=0.04, 95% CI 0.01‐0.11; *P*=.004) functioning scales, with higher means detected in the app arm. After adjusting for baseline scores, only social functioning scales retained the statistical significance (partial η^2^=0.025; *P*=.03). Furthermore, improvements in means over time occurred only in the app arm (Table 5).

**Table 5.** QLQ-C30^a^ functional scales of quality of life according to randomization arm^b^.

	Control^c^, mean (SD)	App^d^, mean (SD)	*P* value^e^	Effect size^f^ (95% CI)	Adjusted *P* value^g^	Adjusted effect size^f^
Physical functioning					—^h^	—
Baseline	80.49 (16.90)	82.60 (16.52)	.38	0.004 (0.00 to 0.04)		
1 year	79.20 (18.06)	81.54 (17.86)	.38	0.004 (0.00 to 0.04)		
Mean change	−2.097 (12.78)	−2.148 (13.40)	.98	0.00 (0.00 to 0.00)		
Role functioning						
Baseline	86.03 (21.65)	89.11 (18.62)	.28	0.006 (−0.00 to 0.04)	—	—
1 year	81.70 (25.20)	89.13 (19.83)	.03	0.026 (0.00 to 0.09)	.11	0.014
Mean change	−4.348 (20.36)	−1.268 (17.69)	.28	0.007 (0.00 to 0.05)	—	—
Emotional functioning					—	—
Baseline	83.93 (15.29)	85.81 (14.84)	.38	0.004 (0.00 to 0.04)		
1 year	82.97 (19.27)	87.72 (14.04)	.06	0.02 (0.00 to 0.08)		
Mean change	−0.815 (16.53)	0.807 (12.17)	.45	0.003 (0.00 to 0.04)		
Cognitive functioning					—	—
Baseline	87.71 (16.08)	88.78 (15.11)	.63	0.001 (0.00 to 0.03)		
1 year	85.90 (19.07)	90.50 (16.00)	.08	0.017 (0.00 to 0.07)		
Mean change	−1.648 (14.50)	0.000 (13.23)	.42	0.004 (0.00 to 0.04)		
Social functioning						
Baseline	90.57 (18.16)	94.72 (12.00)	.06	0.018 (0.00 to 0.07)	—	—
1 year	86.02 (19.24)	93.55 (15.74)	.004	0.044 (0.01 to 0.11)	.03	0.025
Mean change	−4.480 (19.06)	−1.971 (17.18)	.35	0.005 (0.00 to 0.04)	—	—

a QLQ-C30: Quality of Life Questionnaire Core 30.

b Comparison was carried out based on means at individual time points, changes from baseline, and means adjusted to baseline values.

c Control: smoking cessation with written material.

d App: smartphone app–based smoking cessation.

e ANOVA.

f Partial eta-square.

g Analysis of covariance test adjusted for baseline QLQ-C30 functional scales.

h Not applicable.

Of the specific symptom scores, fatigue, pain, insomnia, and financial difficulties were calculated based on the QLQ-C30, while dyspnea at rest and pain in other parts were based on the QLQ-LC13. In the analysis, only insomnia and dyspnea at rest showed differences at baseline, but differences were subtle. At 1 year, fatigue, pain, insomnia, financial difficulties, and pain in other parts showed lower means and symptom burden in the app arm. After adjusting for baseline scores, only pain and final difficulties retained statistical difference (partial η^2^=0.05; *P*=.02; partial η^2^=0.03; *P*=.02). Only the pain score showed a statistically significant difference in mean change (7.065 vs −0.538; partial η^2^=0.026, 95% CI 0.00‐0.09; *P*=.03) between the study arms (Table 6).

We also analyzed specific symptoms from the EQ-5D questionnaire. In the analysis, there was no difference at baseline according to study arm (not shown). At 1 year, we observed improvements in both mobility (partial η^2^=0.031, 95% CI 0.001‐0.09; *P*=.02) and pain or discomfort (partial η^2^=0.049, 95% CI 0.006‐0.12; *P*=.01) with individuals randomized to the app arm, and these retained in the adjusted analysis (partial η^2^=0.037; *P*=.008; partial η^2^=0.026; *P*=.007; Table 7).

**Table 6.** QLQ-C30^a^+QLQ-LC13^b^–specific symptom scores showing significant changes according to randomization arm^c^.

	Control^d^, mean (SD)	App^e^, mean (SD)	*P* value^f^	Effect size^g^ (95% CI)	Adjusted *P* value^h^	Adjusted effect size^g^
Fatigue						
Baseline	24.02 (18.08)	21.56 (19.93)	.36	0.004 (0.00-0.04)	—^i^	—
1 year	26.05 (21.27)	19.59 (17.75)	.03	0.027 (0.00-0.09)	.10	0.015
Mean change	2.031 (15.62)	−0.239 (17.91)	.36	0.005 (0.00-0.04)	—	—
Pain						
Baseline	24.32 (27.62)	18.65 (22.15)	.11	0.013 (0.00-0.06)	—	—
1 year	32.26 (32.68)	16.49 (21.35)	<.001	0.076 (0.02-0.16)	.02	0.05
Mean change	7.065 (26.29)	−0.538 (20.03)	.03	0.026 (0.00-0.09)	—	—
Insomnia						
Baseline	25.25 (27.80)	17.82 (25.19)	.049	0.019 (0.00-0.07)	—	—
1 year	22.58 (27.44)	15.05 (22.26)	.04	0.022 (0.00-0.08)	.25	0.07
Mean change	−1.792 (25.34)	−1.792 (23.24)	>.99	0.00 (0.00-0.00)	—	—
Financial difficulties						
Baseline	10.77 (19.53)	5.94 (17.25)	.06	0.017 (0.00-0.07)	—	—
1 year	13.26 (22.60)	3.94 (14.62)	.001	0.057 (0.01-0.13)	.02	0.03
Mean change	2.151 (18.26)	−0.717 (14.73)	.24	0.007 (0.00-0.05)	—	—
Dyspnea in rest					—	—
Baseline	1.35 (6.60)	4.29 (11.22)	.02	0.025 (0.00-0.008)		
1 year	3.23 (9.91)	2.51 (12.27)	.66	0.001 (0.00-0.03)		
Mean change	1.792 (9.02)	−1.075 (14.29)	.10	0.014 (0.00-0.07)		
Pain in other parts						
Baseline	33.33 (32.97)	26.53 (30.26)	.14	0.012 (0.00-0.06)	—	—
1 year	37.12 (35.53)	24.73 (30.26)	.01	0.035 (0.002-0.10)	.06	0.02
Mean change	2.811 (30.45)	−1.482 (35.30)	.40	0.004 (0.00-0.04)	—	—

a QLQ-C30: Quality of Life Questionnaire Core 30.

b QLQ-LC13: Quality of Life Questionnaire Lung Cancer 13.

c Comparison was carried out based on means at individual time points, changes from baseline, and means adjusted to baseline values.

d Control: smoking cessation with written material.

e App: smartphone app–based smoking cessation.

f ANOVA.

g Partial eta-square.

h Analysis of covariance adjusted for baseline QLQ-C30 symptom scores.

i Not applicable.

**Table 7.** EQ-5D–specific symptom scores and their change between baseline and 1 year according to prespecified classification of the questionnaire according to randomization arm^a^.

	Control^b^, n (%)	App^c^, n (%)	*P* value^d^	Effect size^e^ (95% CI)	Adjusted *P* value^f^	Adjusted effect size^f^
Mobility						
Baseline			.86	0.00 (0.00-0.017)	—^g^	—
No problems	61 (62)	61 (60)				
Some problems	38 (38)	40 (40)				
1 year			.02	0.031 (0.001-0.09)	.008	0.037
No problems	48 (52)	64 (69)				
Some problems	45 (48)	29 (31)				
Pain or discomfort						
Baseline			.46	0.006 (0.00-0.04)	—	—
No pain or discomfort	45 (46)	52 (52)				
Moderate pain or discomfort	51 (52)	58 (48)				
Extreme pain or discomfort	3 (3)	1 (1)				
1 year			.01	0.049 (0.006-0.12)	.007	0.026
No pain or discomfort	36 (39)	55 (59)				
Moderate pain or discomfort	50 (54)	36 (39)				
Extreme pain or discomfort	7 (8)	2 (2)				

a Comparison was carried out based on symptom scales.

b Control: smoking cessation with written material.

c App: smartphone app–based smoking cessation.

d Pearson chi-square test.

e ANOVA.

f Analysis of covariance adjusted for baseline EQ-5D symptom scores.

g Not applicable.

### Frequency of App Use and Its Association to HRQoL

To further support that our observed improvements result from app use, we analyzed the connection between app use and changes in GHS and EQ-5D index scores. We assessed app use by the number of questionnaires the user had completed in the app over the course of the first 24 weeks. There was a statistically significant difference between the mean number of filled questionnaires by those whose EQ-5D index score showed no change or improved (partial η^2^=0.073, 95% CI 0.00‐0.18; *P*=.04; partial η^2^=0.071; adjusted *P*=.04). In the analysis of GHS and app use, nonsignificant trends similar to those of the EQ-5D were observed (Table 8).

**Table 8.** The extent of app use in relation to health-related quality of life^a^.

	Improved	No change	Declined	*P* value^b^	Effect size^b^	Adjusted *P* value^c^	Adjusted effect size^c^
QLQ-C30^d^ GHS^e^ (10% change), n (%)	21 (23)	55 (60)	15 (16.5)	—^f^	—	—	—
Completed questionnaires, mean (SD)	11.14 (14)	6.22 (7)	5.73 (6)	.08	0.056 (0.00-0.16)	—	—
EQ-5D index score (5% change), n (%)	29 (32)	43 (47)	19 (21)	—	—	—	—
Completed questionnaires, mean (SD)	9.86 (11)	7.40 (8)	3.05 (4)	.04	0.073(0.00-0.18)	.04	0.071

a An analysis of EQ-5D index score (5%) or QLQ-C30 (10%) change in the experimental arm. The use of the app was assessed by the number of questionnaires the user had completed in the smoking cessation app over the course of the first 24 weeks.

b One-way ANOVA.

c Analysis of covariance adjusted for age, sex, pack years.

d QLQ-C30: Quality of Life Questionnaire Core 30.

e GHS: global health status.

f Not applicable.

## Discussion

### Principal Findings

To our knowledge, LDCT-SC-FI was the first to report the results of a smartphone app–based smoking cessation in a randomized controlled trial setting among individuals participating in LDCT lung cancer screening. In this study, we investigated the changes in HRQoL in participants undergoing LDCT screening for lung cancer over a 1-year study period using 3 different PROMs. Additionally, we analyzed the effects of smoking cessation as well as the use of our interventional smoking cessation app on HRQoL outcomes. According to our results, neither LDCT screening for lung cancer nor smoking cessation was associated with changes in HRQoL, while the individuals randomized to the Suunta smoking cessation smartphone app reported improved HRQoL mainly by decreased pain and mobility. In general, observed changes in mean values of HRQoL are small, but these, however, fulfill the population norm for significant change (±5%) using the EQ-5D measure. Our results suggest that the positive effects of smoking cessation apps can be comprehensive and may not be limited to cessation only.

In addition to early detection of lung cancer, LDCT screening can have various health effects, which need to be considered when initiating screening programs [2,3,9,11,12]. There is some evidence that LDCT screening may induce negative effects on participants’ HRQoL, especially psychosocial in nature. In most studies, these effects have been short-term and reversed over time [11,13,14]. Our results are in line with these findings, suggesting that lung cancer screening does not negatively impact HRQoL. Since our study had a limited number of participants, we were not able to study the correlation between screening results and HRQoL.

Smoking is the leading risk factor for lung cancer, and smoking cessation clearly prevents the disease as well as improves outcomes [1]. Lung cancer screening programs offer a superb opportunity for smoking cessation interventions and enhance their efficacy [4-6,8]. We have recently shown that smoking cessation can be enhanced 3-fold, and it is of interest if this would translate to alter HRQoL [20]. However, our results showed that smoking cessation does not have an impact on HRQoL or specific scales or symptoms, even though we had hypothesized the contrary. Reflecting on the previous literature, our negative results are not surprising since positive HRQoL effects of smoking cessation might require years to develop [24,27].

### Comparison to Prior Work

Currently, smartphones are ubiquitous and provide an accessible platform for health interventions. In various medical conditions, including cancer, such interventions have shown beneficial effects on HRQoL among other study outcomes [33-35]. In recent years, various smartphone-based methods for smoking cessation have been developed, and some have proven to be effective [18,19]. To our knowledge, these studies have not assessed HRQoL, so our study brings novel information on this topic. Although the Suunta app was effective in smoking cessation and surpassed meta-analysis–based ORs for smoking cessation with mobile apps, we observed no association between cessation at 1 year and HRQoL [18,20]. Interestingly, our study showed that individuals randomized to the Suunta app arm presented with improved overall HRQoL as well as specific scales and symptoms. Improvements in HRQoL seemed to be driven by improvements in social function, mobility, as well as pain. Our findings suggest that the improved HRQoL is a result of a direct effect of Suunta app use, rather than this resulting due to smoking cessation. The effects of mobile health apps on HRQoL have seldom been investigated, and it is possible that other apps would bear similar effects. Furthermore, HRQoL, or happiness, might also be measured with nonvalidated instruments [36]. These are interesting areas of future research.

The theoretical background of the Suunta app includes cognitive behavioral and social cognitive theories and commitment therapy as well as mindfulness. The participants may use the app for goal setting, decision-making, and personal empowerment in smoking cessation as well as for overall management of their health. Functionalities include a weekly symptom questionnaire with personalized feedback, mindfulness practices, self-reflection, and a guided smoking cessation plan. Observed improvements in functional scales might relate to these features, which aim to enhance self-reflection capabilities and induce empowerment.

It is unclear how the Suunta app relays its favorable effects on the pain symptom scores, observed with all 3 PROMs used in this study. To be precise, the pain symptom scores decreased in the app arm over the 1-year study period, whereas in the control arm, they increased. Since the sensation of pain is a complex phenomenon with a psychosocial component, features of the app, specifically mindfulness meditation–based exercises, might alter the overall experience of pain. There are evolving data that mindfulness meditation–based analgesia reduces pain through multiple, unique neural mechanisms and may lead to long-term effects [37]. Our results suggested that the app favorably affected generalized pain rather than regional or nociceptive pain, for example, chest pain and angina, which likely reflects its effect on the psychosocial, and maybe even neurophysiological components of pain.

We also analyzed the extent of app use in relation to HRQoL, and the more frequent use was associated with improved overall HRQoL. This favors the explanation that the features of and adherence to the app are involved in the changes observed. In addition, we have previously shown that adherence to app use was associated with increased smoking cessation [20]. Therefore, the means to increase app adherence could enhance the effects on both smoking cessation and HRQoL. Our results indicate that a mobile app can assist in achieving smoking cessation and retaining or even improving HRQoL during the LDCT screening for lung cancer.

### Strengths and Limitations

Our study has some limitations. The study recruitment occurred only in a single center, and the number of participants was moderate, which might limit the generalizability of the results to a wider screening population. Smoking was assessed by self-reporting without biochemical verification, which may overestimate the number of nonsmokers. Yet, it has previously been shown that self-reported smoking cessation follows closely to biochemically verified data [38]. HRQoL was an exploratory end point of the trial, and analysis was not prespecified in detail, and the results should be interpreted with caution. In addition, HRQoL was analyzed only at 2 time points, which limits the time scope of the analysis. Nevertheless, in the context of the study population, the frequency of HRQoL assessments is reasonable. The credibility of our results is supported by using 3 different PROMs assessing HRQoL, all of which produced similar results. It has been suggested that combining generic HRQoL measures with more condition-specific ones may increase their overall usefulness [39]. In addition, the coverage of HRQoL questionnaires was very high because of good participant adherence. The smartphone app was offered to the controls at 6 months, which might have had an impact on the observed results. However, only 15% (14/93) of the controls reported app use at 1 year, and the observed HRQoL results were very similar to intention-to-treat when excluding these from analysis. As in all clinical trials, there were dropouts in our study. The dropout rate was 7.5%, which is very low compared to smoking cessation studies that often report rates as high as 50%. Furthermore, the dropout rates were very similar in both arms, and it is likely that dropouts have only a marginal impact on observed results.

### Conclusions

We investigated the effect of LDCT lung cancer screening, smoking cessation, and a smoking cessation app on HRQoL. The LDCT-SC-FI trial was the first to investigate a smoking cessation mobile app in the context of LDCT; thus, the presented HRQoL results are unique. We were able to show that randomization to the smartphone app arm, as well as the frequency of app use, was associated with improved HRQoL, while participation in screening or smoking cessation had no effect. According to the study results, the beneficial impact of the app is driven by enhanced social functioning, mobility, and pain compared to controls. Our study suggests that a smartphone app aiming at smoking cessation is an effective intervention in the context of LDCT lung cancer screening since it can both enhance smoking cessation and improve HRQoL.

## Supplementary material

10.2196/81687 Multimedia Appendix 1 Snapshots of the app.

10.2196/81687 Multimedia Appendix 2 Study protocol.

10.2196/81687 Multimedia Appendix 3 LDCT-SC-FI study demographics, and the integrity of the HRQoL questionnaire data.

10.2196/81687 Checklist 1 CONSORT-eHealth checklist (V 1.6.1).

## Abbreviations

CT: computed tomography
EORTC: European Organisation for Research and Treatment of Cancer
ES: effect size
GHS: global health status
HRQoL: health-related quality of life
LDCT: low-dose computed tomography
LDCT-SC-FI: low-dose computed tomography screening for lung cancer combined to different smoking cessation methods in Finland
NELSON: Nederlands-Leuvens Longkanker Screenings Onderzoek
OR: odds ratio
PROM: patient-reported outcome measure
QLQ-C30: Quality of Life Questionnaire Core 30
QLQ-LC13: Quality of Life Questionnaire Lung Cancer 13
QoL: quality of life
