# Supplementary material for: Effect of Lung Cancer Screening, Smoking Cessation, and Cessation Smartphone App to Health-Related Quality of Life Among Heavy Smokers: Randomized Controlled Trial
Source: J Med Internet Res. 2026 Jan 20;28:e81687. doi: 10.2196/81687 (PMC12818497; doi:10.2196/81687)
Supplement: Multimedia Appendix 1 [file jmir-v28-e81687-s001.pdf]

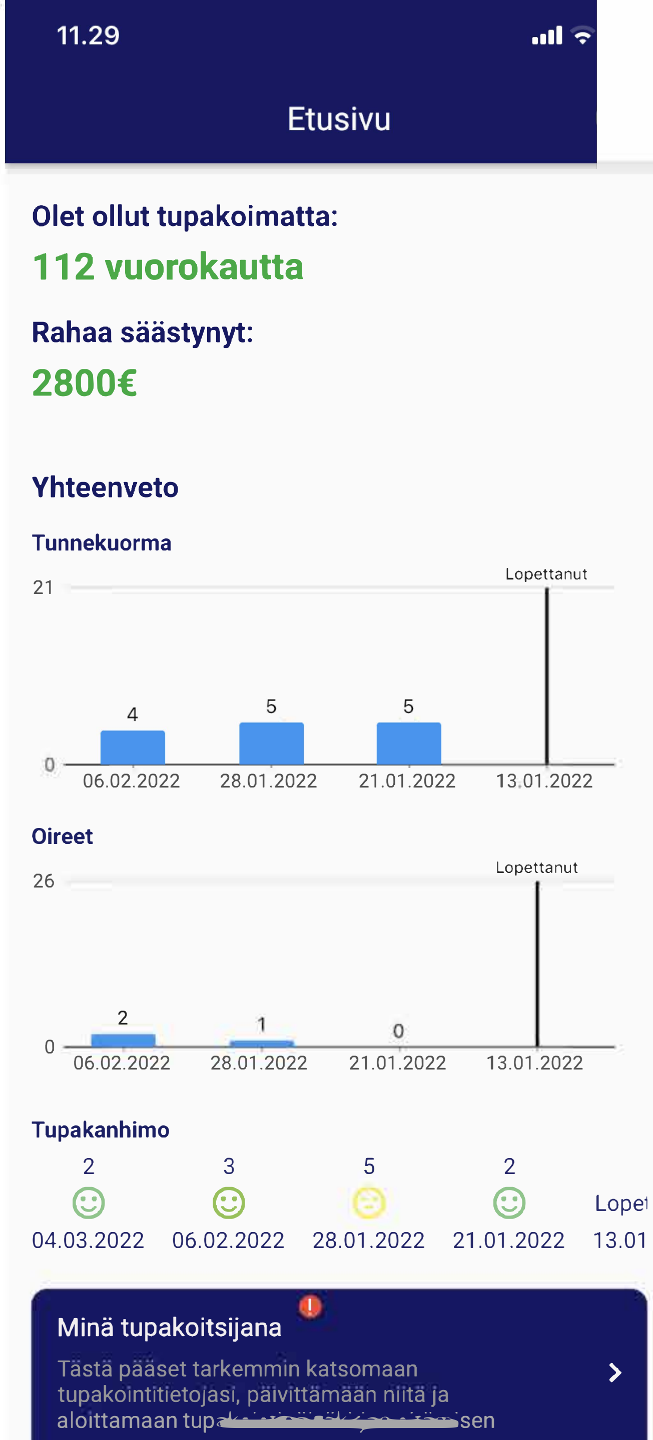
11.29
Etusivu
Olet ollut tupakoimatta:
112 vuorokautta
Rahaa säästynyt:
2800€
Yhteenveto
Tunnekuorma
21
0
Lopettanut
4
5
5
06.02.2022
28.01.2022
21.01.2022
13.01.2022
Oireet
26
0
Lopettanut
2
1
0
06.02.2022
28.01.2022
21.01.2022
13.01.2022
Tupakanhimo
2
3
5
2
Lopet
04.03.2022
06.02.2022
28.01.2022
21.01.2022
13.01
Minä tupakoitsijana
Tästä pääset tarkemmin katsomaan tupakointitietojasi, päivittämään niitä ja aloittamaan tup

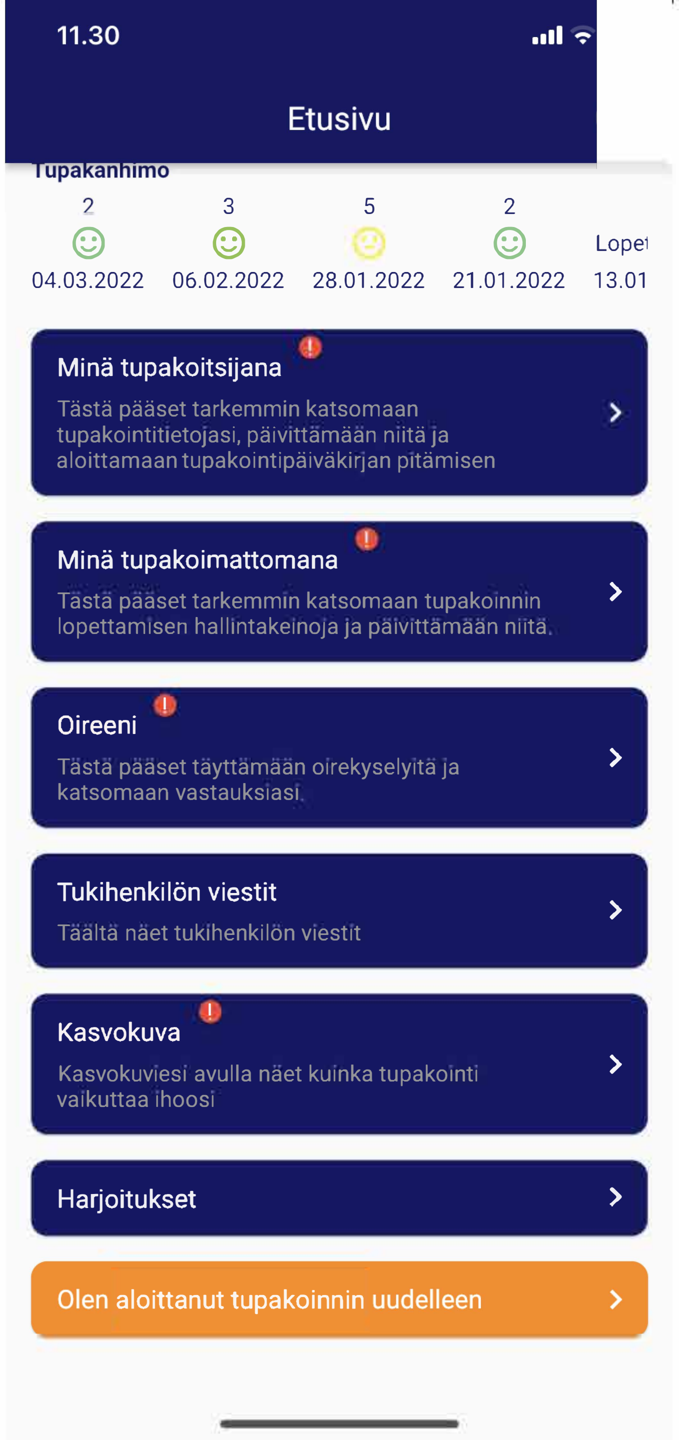
11.30
Etusivu
Tupakanhimo
2
3
5
2
Lopet
04.03.2022
06.02.2022
28.01.2022
21.01.2022
13.01
Minä tupakoitsijana
Tästä pääset tarkemmin katsomaan tupakointitietojasi, päivittämään niitä ja aloittamaan tupakointipäiväkirjan pitämisen
Minä tupakoimattomana
Tästä pääset tarkemmin katsomaan tupakoinnin lopettamisen hallintakeinoja ja päivittämään niitä.
Oireeni
Tästä pääset täyttämään oirekyselyitä ja katsomaan vastauksiasi.
Tukihenkilön viestit
Täältä näet tukihenkilön viestit
Kasvokuva
Kasvokuviesi avulla näet kuinka tupakointi vaikuttaa ihoosi
Harjoitukset
Olen aloittanut tupakoinnin uudelleen

11.31

Oma profiili

Kasvokuva
Kasvokuviesi avulla näet kuinka tupakointi vaikuttaa ihoosi

Profiili

Ilmoitukset

Tupakointitiedot

Askin hinta ja savukkeiden määrä

Käyttöehdot

Tietosuojaseloste

Saavutettavuusseloste

Tietoa

Tutkimuskoodi

Kirjaudu ulos

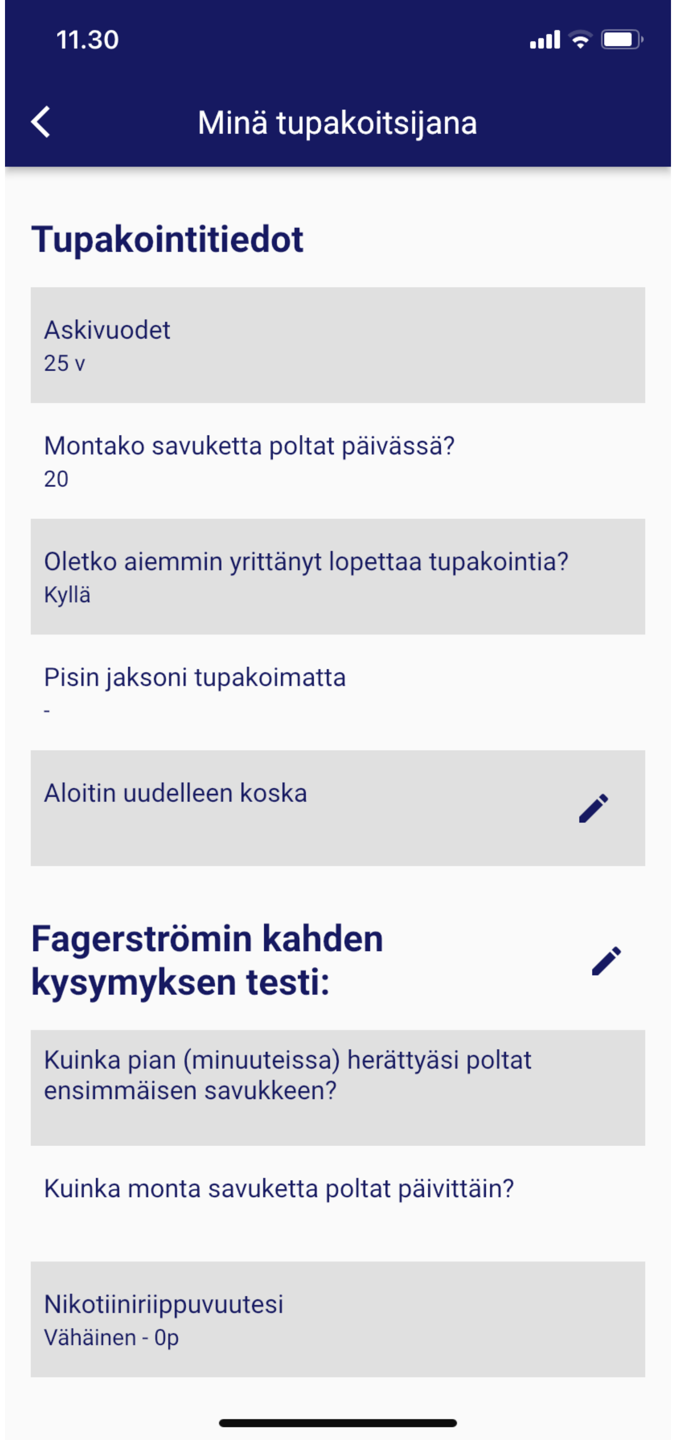
11.30
Minä tupakoitsijana
Tupakointitiedot
Askivuodet
25 v
Montako savuketta poltat päivässä?
20
Oletko aiemmin yrittänyt lopettaa tupakointia?
Kyllä
Pisin jaksoni tupakoimatta
-
Aloitin uudelleen koska
Fagerströmin kahden kysymyksen testi:
Kuinka pian (minuuteissa) herättyäsi poltat ensimmäisen savukkeen?
Kuinka monta savuketta poltat päivittäin?
Nikotiiniriippuvuutesi
Vähäinen - 0p

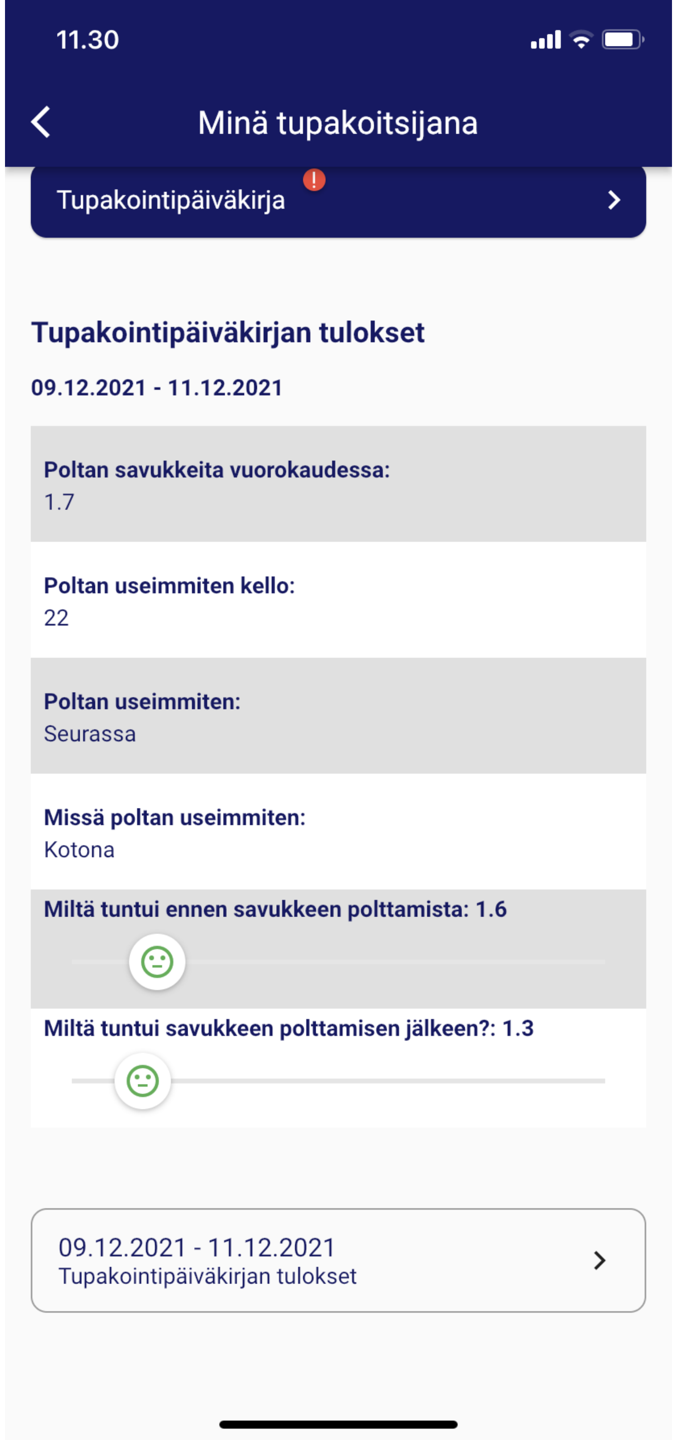
11.30
Minä tupakoitsijana
Tupakointipäiväkirja
Tupakointipäiväkirjan tulokset
09.12.2021 - 11.12.2021
Poltan savukkeita vuorokaudessa:
1.7
Poltan useimmiten kello:
22
Poltan useimmiten:
Seurassa
Missä poltan useimmiten:
Kotona
Miltä tuntui ennen savukkeen polttamista: 1.6
Miltä tuntui savukkeen polttamisen jälkeen?: 1.3
09.12.2021 - 11.12.2021
Tupakointipäiväkirjan tulokset

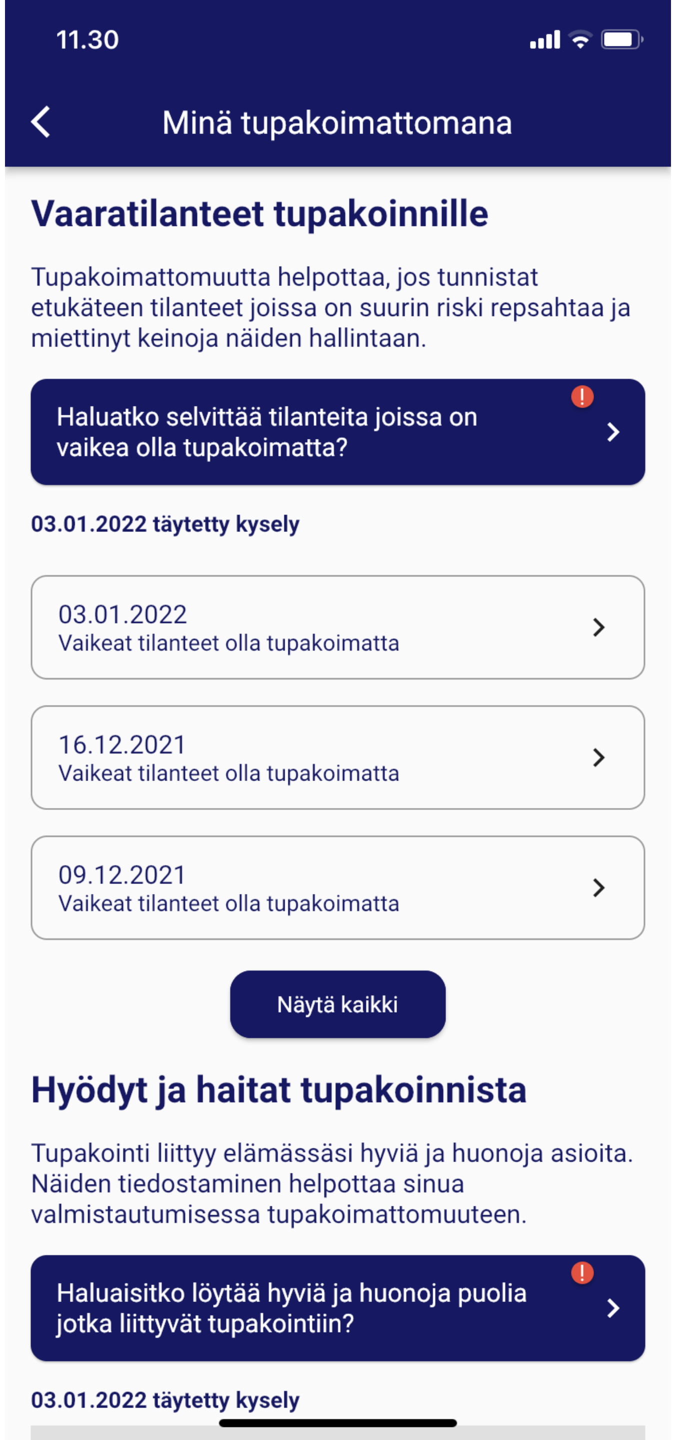
11.30
Minä tupakoimattomana
Vaaratilanteet tupakoinnille
Tupakoimattomuutta helpottaa, jos tunnistat etukäteen tilanteet joissa on suurin riski repsahtaa ja miettinyt keinoja näiden hallintaan.
Haluatko selvittää tilanteita joissa on vaikea olla tupakoimatta?
03.01.2022 täytetty kysely
03.01.2022
Vaikeat tilanteet olla tupakoimatta
16.12.2021
Vaikeat tilanteet olla tupakoimatta
09.12.2021
Vaikeat tilanteet olla tupakoimatta
Näytä kaikki
Hyödyt ja haitat tupakoinnista
Tupakointi liittyy elämässäsi hyviä ja huonoja asioita. Näiden tiedostaminen helpottaa sinua valmistautumisessa tupakoimattomuuteen.
Haluaisitko löytää hyviä ja huonoja puolia jotka liittyvät tupakointiin?
03.01.2022 täytetty kysely

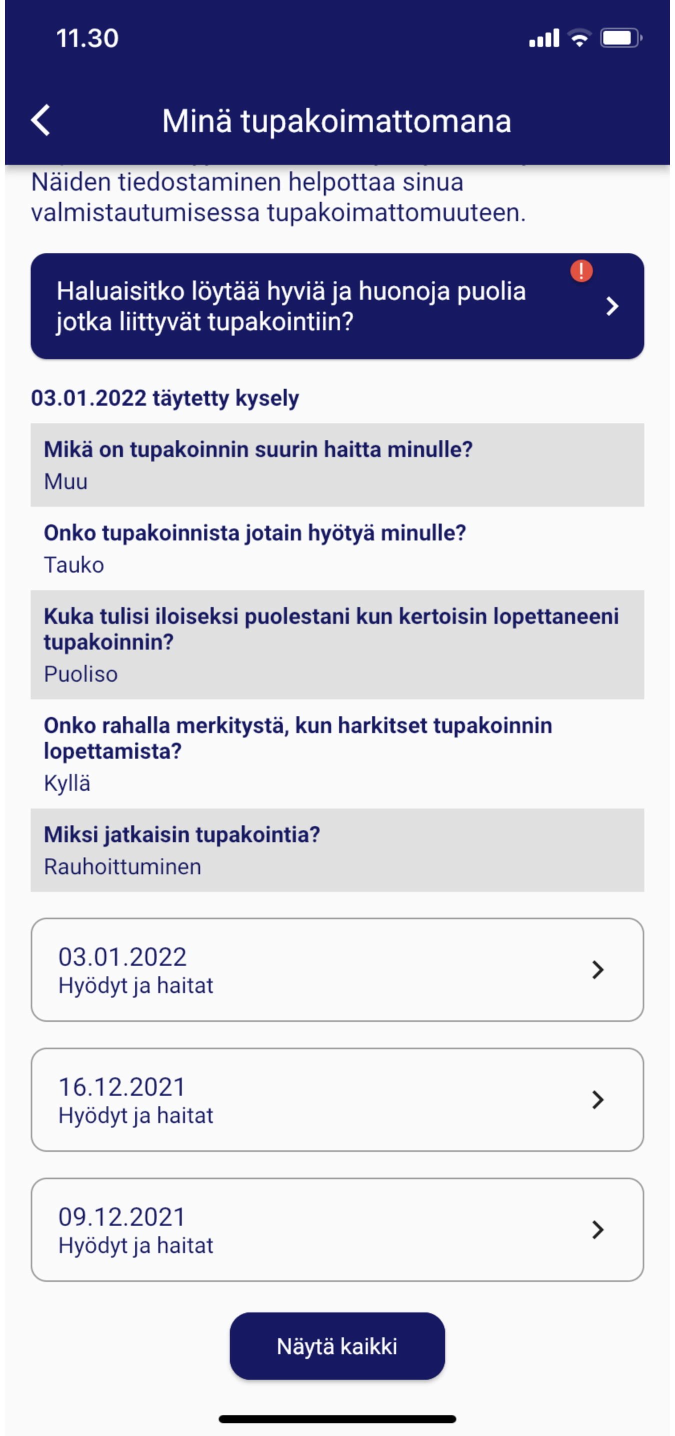
11.30
Minä tupakoimattomana
Näiden tiedostaminen helpottaa sinua valmistautumisessa tupakoimattomuuteen.
Haluaisitko löytää hyviä ja huonoja puolia jotka liittyvät tupakointiin?
03.01.2022 täytetty kysely
Mikä on tupakoinnin suurin haitta minulle?
Muu
Onko tupakoinnista jotain hyötyä minulle?
Tauko
Kuka tulisi iloiseksi puolestani kun kertoisin lopettaneeni tupakoinnin?
Puoliso
Onko rahalla merkitystä, kun harkitset tupakoinnin lopettamista?
Kyllä
Miksi jatkaisin tupakointia?
Rauhoittuminen
03.01.2022
Hyödyt ja haitat
16.12.2021
Hyödyt ja haitat
09.12.2021
Hyödyt ja haitat
Näytä kaikki

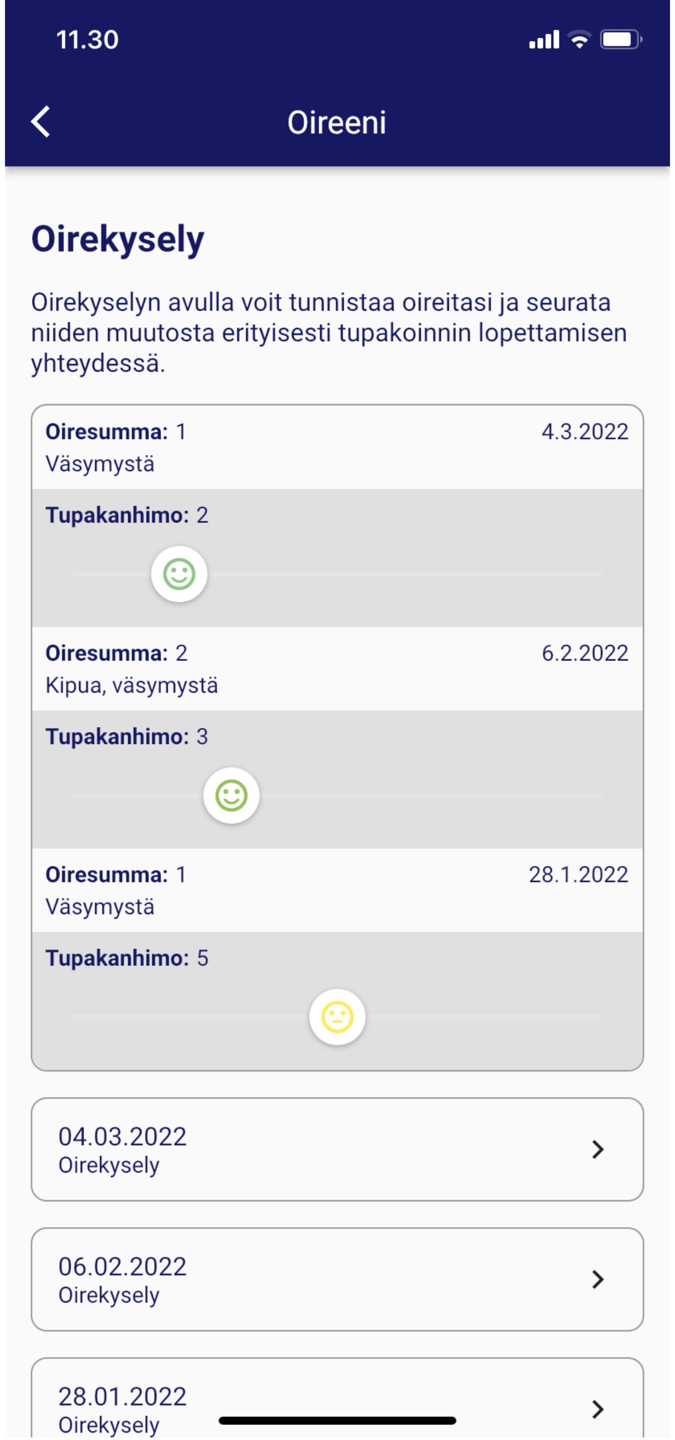
11.30
Oireeni
Oirekysely
Oirekyselyn avulla voit tunnistaa oireitasi ja seurata niiden muutosta erityisesti tupakoinnin lopettamisen yhteydessä.
Oiresumma: 1
4.3.2022
Väsymystä
Tupakanhimo: 2
Oiresumma: 2
6.2.2022
Kipua, väsymystä
Tupakanhimo: 3
Oiresumma: 1
28.1.2022
Väsymystä
Tupakanhimo: 5
04.03.2022
Oirekysely
06.02.2022
Oirekysely
28.01.2022
Oirekysely

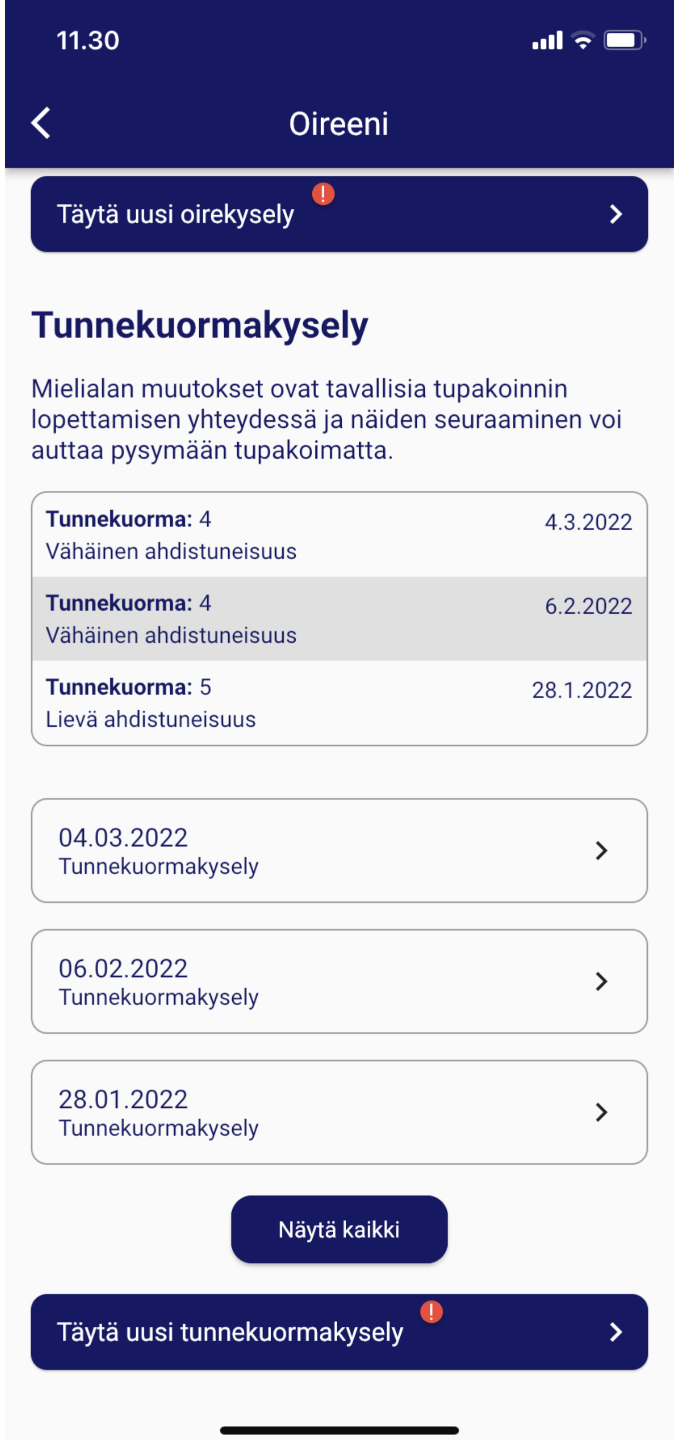
11.30
Oireeni
Täytä uusi oirekysely
Tunnekuormakysely
Mielialan muutokset ovat tavallisia tupakoinnin lopettamisen yhteydessä ja näiden seuraaminen voi auttaa pysymään tupakoimatta.
Tunnekuorma: 4
4.3.2022
Vähäinen ahdistuneisuus
Tunnekuorma: 4
6.2.2022
Vähäinen ahdistuneisuus
Tunnekuorma: 5
28.1.2022
Lievä ahdistuneisuus
04.03.2022
Tunnekuormakysely
06.02.2022
Tunnekuormakysely
28.01.2022
Tunnekuormakysely
Näytä kaikki
Täytä uusi tunnekuormakysely

11.30

Tukihenkilön viestit

Virtuaalitukihenkilön viestit

02.03.2022 kello 12.00
Mikä on ollut vaikeinta tupakoimattomalla tiellä? Haluatko [illegible]

01.03.2022 kello 12.00
Oletko havainnut muutoksia ulkonäössäsi tupakoimattomuuden [illegible]

28.02.2022 kello 12.00
Tupakoimattomana et altista läheisijäsi tupakansavulle ja sen haitallisille [illegible]

27.02.2022 kello 12.00
Kun lopetat tupakoinnin, myös riski sairastua tai kuolla pienenee; [illegible]

26.02.2022 kello 12.00
Onko mielialasi muuttunut tupakoimattoman elämän myötä. Täältä [illegible]

25.02.2022 kello 12.00
Olekto viestinyt tuttavillesi tupakoimattomasta elämästäsi? Saat [illegible]

24.02.2022 kello 12.00
Mikä on parasta tupakoimattomassa elämässä?

23.02.2022 kello 12.00
Rentoutusharjoitukset voivat auttaa [illegible]

11.42 4G

Kasvokuva

Kasvokuva

Tupakointi vaikuttaa ulkoiseen ilmeeseesi ja mm. ihon kuntoon. Kasvokuvien avulla voit vertailla tupakoinnin ja sen lopettamisen vaikutuksia ulkonäköösi.

Pyri ottamaan kuva hyvässä valossa sekä kohtisuoraan kasvojasi kohti. Kuva on tarkoitettu vain omaa seurantaasi varten.

Lisää uusi kasvokuva !

Tupakoi | Lopettanut | Uudelleen aloittanut

Aseta profiilikuvaksi

11.30

Harjoitukset

Täältä löydät rentoutusharjoituksia. Nämä voivat auttaa sinua tupakoinnin lopettamisessa ja tupakoimattomassa elämässä.

Haukottelu ja hengitys

Kädet ylös ja hengitys

Aistiherkitys

Kesäpäivä

Viiden sormen hengitys

Tavoitteen asettaminen
