# Supplementary material for: Effect of Lung Cancer Screening, Smoking Cessation, and Cessation Smartphone App to Health-Related Quality of Life Among Heavy Smokers: Randomized Controlled Trial
Source: J Med Internet Res. 2026 Jan 20;28:e81687. doi: 10.2196/81687 (PMC12818497; doi:10.2196/81687)
Supplement: Multimedia Appendix 2 [file jmir-v28-e81687-s002.pdf]

Low-dose CT screening for lung cancer combined to different smoking cessation methods in Finland (LDCT-SC-FI)

Sponsor Trial Number:

Sponsor: Oulu University Hoispital, Oulu Finland

Clinical Sites:

Oulu University Hospital, Oulu, Finland

Vaasa Central Hospital, Vaasa, Finland

Principal Investigator: Jussi Koivunen, M.D. Ph.D, Sanna Iivanainen, M.D. Ph.D

Co-Investigators: Airi Jartti M.D. Ph.D, Antti Jekunen M.D. Ph.D, Riitta Kaarteenaho M.D. Ph.D, Simo Saarakkala Prof, Tuula Vasankari M.D. Ph.D,

SIGNATURE PAGE

Low-dose CT screening for lung cancer combined to different smoking cessation methods in Finland (LDCT-SC-FI)

Protocol Version 1.2, Feb 14 2023:

Name: ______________________________ Trial Role: _PI_________________________

Signature: ______________________________ Date: __________________________

Address: OYS/syöpäkeskus, Kajaanintie 50, 90220 OYS____

____

Name: ______________________________ Trial Role: __________________________

Signature: ______________________________ Date: __________________________

Address: ____

____

Name: ______________________________ Trial Role: __________________________

Signature: ______________________________ Date: __________________________

Address: ____

____

AMENDMENTS

The following amendments and/or administrative changes have been made to this protocol since the implementation of the first approved version:

Amendment number	Date of amendment	Protocol version number	Type of amendment	Summary of amendment
1.	25.5.2022	1.1	Amendment	1. Correction of differences between summary and eligibility section (sections 1. and 4.1)<br>2. Description of radiation exposure in LDCT, and risk-benefit ratio of LDCT(section 7.3)<br>3. Removal of incorrect wording related to aother trial (section 11.2 and 12.4)<br>4. Correction of sample size calculations to detect 15% difference (75 vs 90%) between the study groups (section 1. and 17.2.1)
2.	14.2.2023	1.2	Amendment	1. Offer a possibility for the study subjects randomized to standard-of-care smoking cessation to use the smoking cessation application after 6 months o follow-up.<br>2. Conversion of specifity of LDCT screening to a exploratory end-point<br>3. Introduction of two new secondary end-points<br>- Percentage of subjects who have reduced the amount of smoked cigrattes/d at 3 & 6 months after inclusion<br>- Magnitude and percentage of reduction in cigarettes consumed/d at 3 & 6 month after inclusion

1. PROTOCOL SUMMARY

This is a randomized phase II trial investigating different smoking cessation methods in study subjects undergoing lung cancer screening with low-dose CT (LDCT). The study also evaluates feasibility and outcomes of LDCT screening and potential biomarkers and AI-based evaluation on CT-scans. The study subjects fulfilling I/E-criteria are randomized in 1:1 fashion to yearly LDCT for two rounds with standard smoking cessation (control) or same LDCT approach combined to mobile application based smoking cessation (intervention).

Figure 1. Study flow-chart

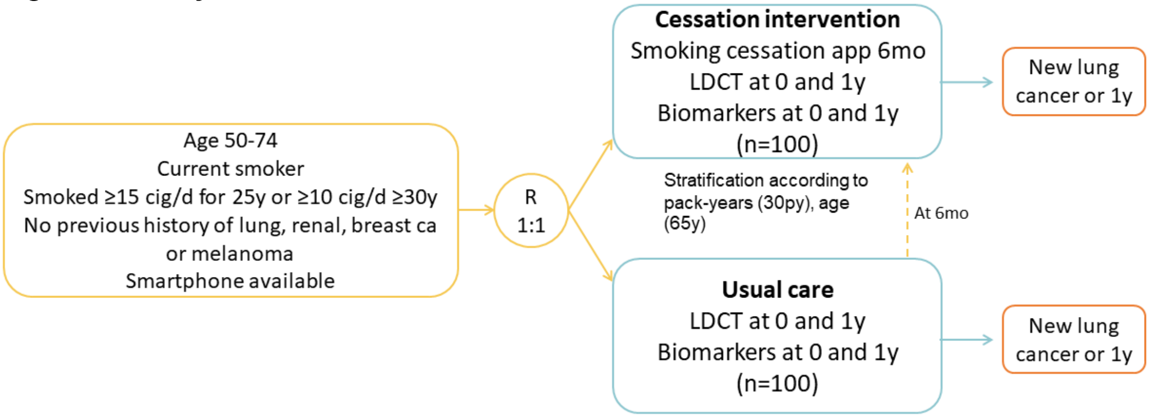

PRIMARY END-POINT
Number of active smokers at 3 and 6 months

SECONDARY END-POINTS
The number of subject who have reduced smoking and amount of reduction
Sensitivity of LDCT
Overall costs and feasibility of LDCT

Primary Objective

- To determine the efficiency of different smoking cessation methods

Secondary Objectives

- To evaluate efficiency of different smoking cessation methods in reduction of smoking
- To assess the sensitivity, and positive predictive value of CT-screening
- To evaluate costs related to CT screening per patient per year

Exploratory Objectives

- To evaluate specifity of CT-screening
- To evaluate lung cancer incidence (stage specific) and survival
- To evaluate the incidence of other important lung disease such as interstitial and fibrotic lung diseases, emphysema, bronchiectasis
- To collect the number of additional CT-scans, PET-CT scans, bronchoscopies, needle biopsies, and their results (positive vs. negative for cancer) initiated based on screening CT-findings
- To evaluate the percentage of subjects identified through newspaper advertisement and referral from primary care physician or occupational health physician
- To Evaluate Quality of life (QoL)
- To collect spectrum and grading of symptoms by electoronic patient reported outcomes (ePRO)
- To evaluate correlation of ePRO symptoms to CT screen results
- To collect blood samples for potential biomarker analysis such as circulating tumor DNA (ctDNA)

- To evaluate artificial intelligence (AI)-based assessment of the LDCT scans

Outcome Measures

Primary outcome measure(s)

- The percentage of subjects who are actively smoking at 3 months after inclusion between the study arms
- The percentage of subjects who are actively smoking at 6 months after inclusion between the study arms

Secondary outcome measures

- The percentage of subjects who have reduced the amount of smoked cigarettes/d at 3 and 6 months after inclusion between the study arms
- The magnitude and percentage of reduction in cigrattes consumed/d at 3 and 6 months after inclusion between the study arms
- Sensitivity, specificity and positive predictive value of CT-screening in the whole cohort
- Costs related to CT screening including additional investigations

Exploratory outcome measures

- Specificity of CT-screening in the whole cohort
- Lung cancer incidence (stage specific) and survival
- Incidence of other clinically important lung disease in LDCT scans
- Number of additional CT-scans, PET-CT scans, bronchoscopies, needle biopsies, and their results (positive vs. negative for cancer) initiated based on screening CT-findings
- Percentage of subjects identified through newspaper advertisement and referral from primary care physician or occupational health physician
- Quality of life (QoL)
- Spectrum and grading of ePRO symptoms
- Correlation of ePRO symptoms to CT screen results
- Biomarker analysis of blood such as ctDNA
- AI-based evaluation of LDCT scans

Trial Subject Population
The subjects at age of 50-74 with significant smoking history and current smoking status will be included in the study.

Sample Size
A maximum of 200 subject will be included in the study.

Eligibility

Inclusion Criteria:
1. Able to provide written informed consent
2. Age between 50-74
3. Smoked ≥ 15 cigarettes/day for ≥ 25 years or smoked ≥ 10 cigarettes/day for ≥ 30 years and are active smokers (smoking during the last two weeks)
4. Access to a smartphone (iPhone or Android)

Exclusion Criteria:
1. A moderate or bad self-reported health; e.g. unable to climb two flights of stairs
2. Body weight ≥ 140 kilogram
3. Current or past melanoma, lung, renal or breast cancer
4. A chest CT examination less than one year before inclusion
5. Has known psychiatric or substance abuse disorders that would interfere with cooperation with the requirements of the trial
6. Subject is unwilling or unable to comply with treatment and trial instructions
7. Any condition that study investigators consider an impediment to safe trial participation

Trial Duration
The enrollment period is estimated to be 6 months. For each subject, the trial will consist of a screening (maximum of four weeks), and intervention period (~1y +/- 3mo). Survival and lung cancer data will be collected beyond the subject's active participation in the trial. The collection of the survival and lung cancer data of a subject is limited to 3 years from the inclusion.

Statistical Methods
Eligible subjects are randomized (1:1) with stratification according to pack years (<30py or ≥30py), and age (<65 or ≥65v) to smartphone based smoking cessation and control arm.

The study is powered (80%) with 155 subjects to detect 15% difference in (75 vs. 90%) in the number of active smokers at three and six months after inclusion with 90% confidence. With the expected dropout rate, the sample size is adjusted to 200.

Intervention Schedule
The intervention period will initiate from randomization. At the randomization visit, all patients will be given written smoking cessation material and blood samples will be collected for biomarker analysis. The patients randomized to intervention arm will be given access to smartphone-based smoking cessation application with user training. The LDCT will be scheduled preferably within six weeks of randomization. After the LDCT, the patient will be informed of the results by mail. If no further procedures are required,

next LDCT will be scheduled for 1y +/- two months). With intermediate LDCT results, follow-up scan will be ordered at three months. With positive LDCT results, the patient will be referred to pulmonologist. At three and six months (+/- one month) after randomization, patient is called to evaluate whether they are active smokers and is they have quitted what is the date of smoking cessation. After the second round LDCT, the subject is scheduled a study visit in which LDCT results are communicated to the subject. In the case ot negative LDCT results, patien enter survival follow-up. With intermediate results, new LDCT will be ordered at 2 or 12months, and positive results will result in referral to pulmonologist. The survival follow-up period can last up to 3 years from inclusion.

Study Assessments

The study assessments for the trial are shown in Table 1.

Table 1. Schedule of Assessments

	Screening (<4wks)	0wks	0-6wks	T3mo (+/- 1mo)	T6mo (+/- 1mo)	T1y (+/- 2mo)	T3y (+/- 2mo)
**Informed consent**	X						
**Health information**	X					X	X
**Smoking status**	X			X	X	X	
**QoL assessment (EQ-5D, QLQ-C30+LC13)**			X			X	
**LDCT**			X			X	
**Blood sampling**[1]			X			X	
**Randomization**		X					
**Smoking cessation app (investigational arm)**		X	X	X	X		
**Smoking cessation mat (control arm)**		X			X (optional)	X (optional)	
**Survival and lung cancer status**						X	X
**CRF forms to be completed**	HI	Rand	LDCT 0<br>LDCT 0+<br>EQ-5D/QLQ<br>Bio 0	Smo 3kk	Smo 6kk	HiFu 1v<br>Smo 1v<br>EQ-5D/QLQ<br>LDCT 1v<br>LDCT 1v+<br>Bio 1v<br>Sur 1v	HiFu 3v<br>Sur 3v

[1]Blood sampling for biomarker analysis (serum, plasma, and ctDNA)

ABBREVIATIONS

AE	Adverse event
CT	Computer tomography
D	Day
DLT	Dose limiting toxicity
ECOG	Eastern Cooperative Oncology Group
eCRF	Electronic case report form
EU	European Union
IEC	Independent Ethics Committee
LDCT	Low-dose computer tomography
Mo	Month
NCI-CTCAE	National Cancer Institute-Common Terminology Criteria for Adverse Events
NSCLC	Non-Small Cell Lung Cancer
OS	Overall survival
SAE	Severe adverse event
SCLC	Small Cell Lung Cancer
SOC	Standard of care
SUSAR	Suspected Unexpected Serious Adverse Reaction
Wk	Week

Table of Contents

SIGNATURE PAGE **2**

AMENDMENTS **3**

1. PROTOCOL SUMMARY ***4***

Primary Objective 4
Secondary Objectives 4
Exploratory Objectives 4
Outcome Measures 5
Trial Subject Population 6
Sample Size 6
Eligibility 6
Inclusion Criteria: 6
Exclusion Criteria: 6
Trial Duration 6
Statistical Methods 6
Intervention Schedule 6
Study Assessments 7

ABBREVIATIONS **9**

1. BACKGROUND AND RATIONALE ***13***

1.1 Lung cancer **13**

1.2 Lung cancer screening **13**

1.3 Smoking cessation in relation to lung cancer screening **13**

2. OBJECTIVES ***14***

2.1 Objectives **14**

2.1.1 Primary Objective 14
2.1.2 Secondary Objectives 14
2.1.3 Exploratory Objectives 14
2.1.4 Outcome Measures 14

3. TRIAL DESIGN ***15***

4. ELIGIBILITY ***16***

4.1 Inclusion and Exclusion Criteria **16**

4.1.1 Inclusion Criteria 16
4.1.2 Exclusion Criteria 16
4.1.3 Specific Additional Exclusion Criteria 16

5. SCREEN AND CONSENT ***16***

5.1 Informed Consent **16**

5.2 Screening **16**

6. TRIAL ENTRY ***17***

7. EXPERIMENTAL PROCEDURES *17*
7.1 Randomization **17**
7.2 Smoking cessation approaches **17**
7.2.1 Smoking status follow-up 17
7.3 LDCT **17**
7.4 Biomarkers **18**
7.5 Lung cancer status and survival follow-up **18**
8. SCREENING RESULT COMMUNICATION TO THE SUBJECT *19*
9. QUALITY REQUIREMENTS AND CONTROL MEASURES FOR LDCT *20*
9.1 Qualifications of Personnel **20**
9.1.1 Qualifications of Study Physicians 20
9.1.2 Qualifications of Study Nurses 20
9.2 CT Equipment Certification and Qualifications **20**
10. Smoking Cessation Application *20*
10.1 Cessation Application Description and ePRO questionnaire **20**
10.2 Software Development **20**
10.3 Chacterization of Properties **20**
11. CONCOMITANT INTERVENTIONS *21*
11.1 Prohibited Interventions **21**
11.2 Trial Subject Withdrawal/Discontinuation **21**
12. ADVERSE EVENT REPORTING *21*
12.1 Definition of Adverse Event **21**
12.2 Definition of Serious Adverse Event **21**
12.3 Characterizing Adverse Events by Attribution and Severity **22**
12.3.1 Direct and Indirect AEs in Screening Imaging Trials 22
12.4 Serious Adverse Events **22**
10.5 AE/SAE Recording and Reporting Period **23**
13. Device Safety *23*
13.1 Safety Plan **23**
13.1.1 Risks Associated with the smoking cessation application 23
13.2 Management of Participants Who Experience Adverse Device Events **23**
13.2.1 Safety Parameters and Definitions 23
13.2.2 Follow-up of Participants after Adverse Device Effects 24
13.2.3 Adverse Device Effects That Occur after the Adverse Device Effect Reporting Period 24
14. DATA HANDLING AND RECORD KEEPING *24*
14.1 Data Collection **24**

14.2 Device Data **25**

14.3 Archiving **25**

15. QUALITY MANAGEMENT ***25***

15.1 Site Set-up and Initiation **25**

15.2 On-Site Monitoring **26**

15.3 Audit and Inspection **26**

15.4 Notification of Serious Breaches **26**

16. END OF TRIAL DEFINITION ***26***

17. STATISTICAL CONSIDERATIONS ***27***

17.1 Trial Population **27**

17.2 Analysis of Outcome Measures **27**

17.2.1 Primary Outcomes 27

17.2.2 Secondary Outcomes 27

17.2.3 Exploratory Outcomes 27

17.3 Analysis of Efficacy Parameters **28**

17.4 Planned Interim Analysis **28**

18. ACCESS TO SOURCE DATA AND RELATED DOCUMENTS ***28***

19. TRIAL ORGANIZATIONAL STRUCTURE ***28***

19.1 Sponsor **28**

19.2 Finance **28**

20. ETHICAL CONSIDERATION ***28***

21. CONFIDENTIALITY AND DATA PROTECTION ***29***

22. PUBLICATION POLICY ***29***

20. REFERENCE ***30***

APPENDIX 1 - WMA DECLARATION OF HELSINKI ***31***

APPENDIX 2 - DEFINITION OF ADVERSE EVENTS ***35***

APPENDIX 3 - COMMON TOXICITY CRITERIA GRADINGS ***36***

1. BACKGROUND AND RATIONALE

1.1 Lung cancer

Lung cancer is the leading cause of cancer-related mortality worldwide. The poor survival prognosis is largely due to a diagnosis at an advanced stage. However, early-stage non-small cell lung cancer (NSCLC) can be treated with surgical resection, stereotactic radiotherapy, or chemoradiotherapy with curative intent and, thus, has a more favorable prognosis.

The most important risk factor for lung cancer is tobacco smoking. Smoking is associated with approximately 85% of all lung cancer cases in Europe. Increasing age and cumulative exposure to tobacco smoke are the two most common risk factors for lung cancer. Smoking related duration-specific risks for lung cancer increase steadily but are the most significant beyond 20 years of smoking.

1.2 Lung cancer screening

Previous trials have shown that lung cancer stage distribution or prognosis cannot be altered with chest x-ray screening. The National Lung Screening Trial (NLST) has demonstrated that screening among adults at high risk using low-dose computed tomography (LDCT) reduced mortality from lung cancer by 20% as compared to individuals who received a standard chest X-ray. Although, these low-dose CT screening benefits were balanced by a very high frequency of false positives; 96% of the positive findings were false-positive results and 23% of all screening test were false-positive results. A recent European LDCT vs. no screening study (NELSON) has demonstrated similar reduction in lung-cancer mortality (24%). Compared to NLST, NELSON study used volumetric assessment of the lesions and this reduced the false-positive rate dramatically to 56.5%. The most important inclusion criteria for NLST and NELSON trial are presented in the Table 2.

Based on these encouraging results, LDCT has been adopted in the USA. Furthermore, many European countries are evaluating implementation of LDCT to national screening programs or initiating pilots to assess feasibility and costs of LDCT screening. However, this is not the case in Finland and LDCT screening has not been evaluated in the country.

Table 2. Age and smoking history related inclusion criteria of NLST and NELSON trials

	NLST	NELSON
**Age (y)**	55-74	50-74
**Smoking history**	30 ≥ pack-years of cigarette smoking history	Smoked ≥15 cigarettes/d for ≥ 25 years
	Former smokers: quit smoking ≤ 15 years	Smoked ≥10 cigarettes/d for ≥ 30 years

1.3 Smoking cessation in relation to lung cancer screening

Smoking cessation is recommended to be incorporated to lung cancer screening programs. LDCT screening has been shown to serve as a ”teachable moment” that encourages cessation in the high risk smoking population.

Smoking cessation in addition to lung cancer screening could further reduce lung cancer-related mortality beyond the effects of LDCT screening alone. Furthermore, tobacco-related healthcare costs are not only limited to lung cancer diseases and smoking is an important risk factor for e.g. numerous

other cancers and cardiovascular diseases. Therefore, smoking cessation has an extensive health and economical impact.

2. OBJECTIVES

2.1 Objectives

2.1.1 Primary Objective

- To determine the efficiency of different smoking cessation methods

2.1.2 Secondary Objectives

- To evaluate efficiency of different smoking cessation methods in reduction of smoking
- To assess the sensitivity and positive predictive value of CT-screening
- To evaluate costs related to CT screening

2.1.3 Exploratory Objectives

- To evaluate specifity of CT-screening
- Lung cancer incidence (stage specific) and survival
- Number of additional CT-scans, PET-CT scans, bronchoscopies, needle biopsies, and their results (positive vs. negative for cancer) initiated based on screening CT-findings
- Percentage of subjects identified through newspaper advertisement and referral from primary care physician or occupational health physician
- Quality of life (QoL)
- Spectrum and grading of ePRO symptoms
- Correlation of ePRO symptoms to CT screen results
- Biomarker analysis of blood such as ctDNA
- AI-based evaluation of LDCT scans

2.1.4 Outcome Measures

Primary outcome measures

- The percentage of subjects who are actively smoking at 3 months after inclusion between the study arms
- The percentage of subjects who are actively smoking at 6 months after inclusion between the study arms

Secondary outcome measures

- The percentage of subjects who have reduced the amount of smoked cigarettes/d at 3 and 6 months after inclusion between the study arms
- The magnitude and percentage of reduction in cigrattes consumed/d at 3 and 6 months after inclusion between the study arms
- Sensitivity and positive predictive value of CT-screening in the whole cohort
- Costs related to CT screening including additional investigations per patient per year

Exploratory outcome measures

- Specificity of CT-screening in the whole cohort
- Lung cancer incidence (stage specific) and survival
- Number of additional CT-scans, PET-CT scans, bronchoscopies, needle biopsies, and their results (positive vs. negative for cancer) initiated based on screening CT-findings

- Percentage of subjects identified through newspaper advertisement and referral from primary care physician or occupational health physician
- Quality of life (QoL)
- Spectrum and grading of ePRO symptoms
- Correlation of ePRO symptoms to CT screen results
- Biomarker analysis of blood such as ctDNA
- AI-based evaluation of LDCT scans

3. TRIAL DESIGN

This is a prospective randomized two arm Phase II trial to evaluate efficiency of different smoking cessation methods in subjects undergoing LDCT screening for lung cancer (Figure 1). For each subject, the trial will consist of a screening period (maximum four weeks) and intervention phase (for ~1y +/- 3 months). Survival data will be collected beyond the subject's active participation in the trial. The collection of the survival data of a subject is limited to 3 years from the randomization.

The primary objective of the trial is to assess the percentage of subjects who are actively smoking at 3 and 6 months after randomization.

Figure 2. Study flow-chart

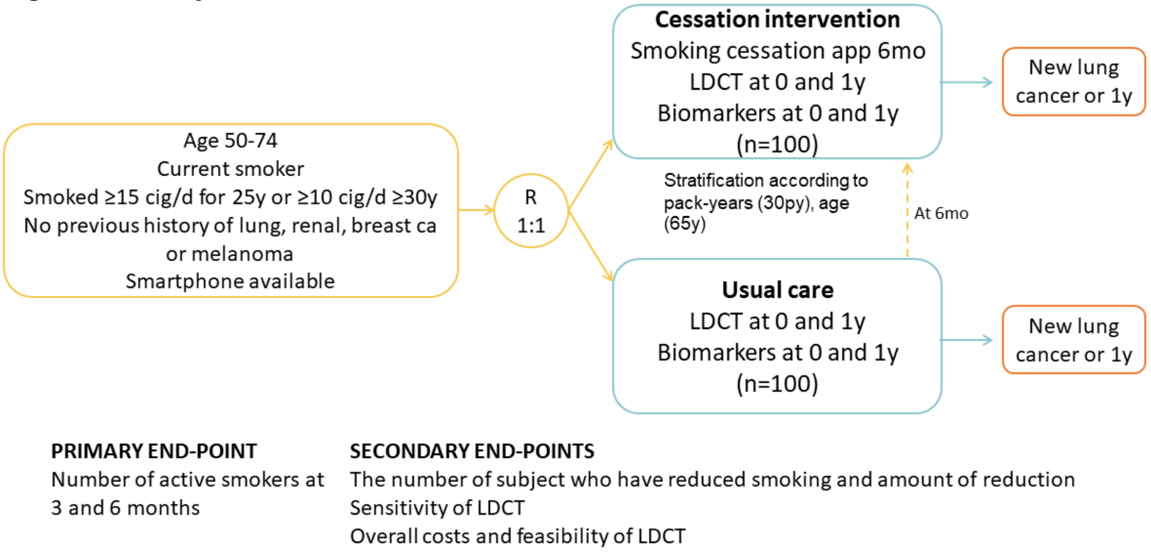

4. ELIGIBILITY

4.1 Inclusion and Exclusion Criteria

4.1.1 Inclusion Criteria

1. Able to provide written informed consent
2. Age between 50-74y
5. Sm Smoked ≥ 15 cigarettes/day for ≥ 25 years or smoked ≥ 10 cigarettes/day for ≥ 30 years and are active smokers (smoking during the last two weeks)
3. Access to a smartphone (iPhone or Android)

4.1.2 Exclusion Criteria

1. A moderate or bad self-reported health; e.g. unable to climb two flights of stairs
2. Body weight ≥ 140 kilogram
3. Current or past melanoma, lung, renal or breast cancer
4. A chest CT examination less than one year before inclusion
5. Has known psychiatric or substance abuse disorders that would interfere with cooperation with the requirements of the trial
6. Subject is unwilling or unable to comply with treatment and trial instructions
7. Any condition that study investigators consider an impediment to safe trial participation

4.1.3 Specific Additional Exclusion Criteria

None

5. SCREEN AND CONSENT

5.1 Informed Consent

Informed consent is obtained by qualified trial investigators according to the standard practice of study site.

5.2 Screening

During the screening period, a subject's eligibility for the trial is determined by evaluation of the exclusion and inclusion criteria. Subjects will undergo a medical history review as part of the screening assessment.

The parameters listed below are collected and recorded into the CRF during the screening period. All procedures must occur within 4 weeks.

- Demographic data (age, race, and sex)
- Inclusion/Exclusion criteria
- Medical history and concomitant medications and interventional procedures
- Smoking history
- QoL with EQ-5D
- Blood sampling for biomarkers

6. TRIAL ENTRY

Once the subject has signed informed consent, a subject number will be assigned. After the eligibility check and the enrolment confirmation, any change in the subject eligibility criteria needs to be reported in the CRF and the suitability of the subject to enter the study treatment must be carefully evaluated by the investigator. Once assigned, the subject numbers for any screening failures non-evaluable, or discontinued subjects will not be re-used. Intend-to-treat (ITT) population will be characterized by subject who fulfill the inclusion and exclusion criteria and receive smoking cessation intervention in form of either written material or mobile application.

7. EXPERIMENTAL PROCEDURES

7.1 Randomization

Eligible individuals with written informed consent are randomized (1:1) with stratification according to pack years (<30py or ≥30py), and age (<65 or ≥65v) to experimental smartphone smoking cessation application or control arm.

7.2 Smoking cessation approaches

In the control arm, the subjects will be given standard written materials on smoking cessation. After six months of follow-up, the subjects in the control arm will be offered access to the smoking cessation application.

In the experimental arm, the subjects will be given access to a smartphone smoking cessation application in addition to standard written materials on smoking cessation.

7.2.1 Smoking status follow-up

The smoking status is verified at the baseline during the study visit (T0)

The smoking status (cessation and reduction in smoking) will be verified by a phone call to the study subject at 3 and 6 months (-/+2 weeks). Furthermore, the smoking status is evaluated during the study visit at 1y (+/- 3mo) (T1).

In the experimental arm, the smoking status information obtained by the study personel can be verified from the data generated by smoking cessation application.

7.3 LDCT

All the study subjects will undergo LDCT screening within six weeks of randomization. After the LDCT, the patient will be informed of the results by mail. If no further procedures are required, next LDCT will be scheduled for 1y +/- two months). With intermediate LDCT results, follow-up scan will be ordered at three months. With positive LDCT results, the patient will be referred to pulmonologist. After the second round LDCT, the subject is scheduled a study visit in which LDCT results are communicated to the subject. In the case ot negative LDCT results, patien enter survival follow-up. With intermediate results, new LDCT will be ordered at 2 or 12months, and positive results will result in referral to pulmonologist. Nodule detection algorithm is presented in Figure 3.

Radiation exposure of single LDCT (effective dose) is 1.6-2.4 mSv, which corresponds to about ½-1v background radiation exposure. During its lifetime, humans are exposed to ionizing radiation from both

natural and artificial sources and the average effective dose received by Finns in 2018 is 5.9 mSv, of which about two thirds (4 mSv) is caused by radon. The additional risk caused by radiation from CT scans performed during the study, ie the risk of cancer, is low. Randomized evidence suggest that annual LDCT in the investigated population decreases lung cancer and all cause mortality and therefore benefits of LDCT clearly overweigth the potential harms.

7.4 Biomarkers

The study subjects will undergo blood sampling for experimental biomarker analysis such as circulating tumor DNA at baseline and 1y.

7.5 Lung cancer status and survival follow-up

The lung cancer status and stage at diagnosis will be followed during the intervention period. Furthermore, the status will be updated from electronic patients records and/or phone call up to 3y from randomization.

Figure 3. Nodule detection algorithm (based on NELSON study protocol)

Screen round 1: baseline		Screen round 1: follow-up scan		Screen round 2-3		Screen round 4	
Screening result	Protocol	Screening result	Protocol	Screening result	Protocol	Screening result	Protocol
NEGATIVE<br>- NODCAT I<br>- NODCAT II	Screening next round	NEGATIVE<br>- GROWCAT A<br>- GROWCAT B	Screening next round	NEGATIVE<br>- new and NODCAT I<br>- GROWCAT A	Screening next round	NEGATIVE<br>- NODCAT I<br>- NODCAT II<br>- GROWCAT A	End of screening
INDETERMINATE<br>- NODCAT III	Follow-up scan after 3 months	POSITIVE<br>- GROWCAT C	Referral pulmonologist	INDETERMINATE<br>- GROWCAT B<br>- new and NODCAT II	Follow-up scan after 12 months	INDETERMINATE<br>- GROWCAT B	Follow-up scan after 12 months
POSITIVE<br>- NODCAT IV	Referral pulmonologist			INDETERMINATE<br>- new and NODCAT III	Follow-up scan after 6-8 weeks	INDETERMINATE<br>- new and NODCAT III	Follow-up scan after 6-8 weeks
				POSITIVE<br>- GROWCAT C<br>- new and NODCAT IV	Referral pulmonologist	POSITIVE<br>- NODCAT IV<br>- GROWCAT C	Referral pulmonologist

NODULE CATEGORy based on volume

NODCAT I	nodule with benign characteristics, as fat/benign calcifications
NODCAT II	solid nodules with a volume of <50 mm^3<br>pleural-based solid nodules with a minimal diameter of <5 mm<br>non-solid component partial solid nodule with a mean diameter of <8 mm<br>non-solid nodules with a mean diameter of <8 mm
NODCAT III	solid nodules with a volume of 50-500 mm^3<br>pleural-based solid nodules with a minimal diameter of 5-10 mm<br>solid nodule with a non-solid component with a mean diameter of ≥8mm
NODCAT IV	solid nodules with a volume of >500 mm^3<br>pleural-based solide nodule with a minimal diameter of >10 mm<br>solid component in a partial solid nodule with a volume of >500 mm^3

NODULE CATEGORY based on volumedoublingtime (growth)

GROWCAT A	volumedoublingtime >600 dagen
GROWCAT B	volumedoublingtime 400-600 dagen
GROWCAT C	volumedoublingtime <400 dagen<br>new solid component in previously exisiting non-solid nodule

At three and six months (+/- one month) after randomization, patient is called to evaluate whether they are active smokers and is they have quitted what is the date of smoking cessation.

The survival follow-up period can last up to 3 years from inclusion

8. SCREENING RESULT COMMUNICATION TO THE SUBJECT

The results of screening will be communicated in writing to the participants. The letters of explanation will be issued withing four weeks of performance of the screening study.

The participants with positive finding will be directly referred to a pulmonologist.

The participants with intermediate findings will undergo another LDCT in a scheduled manner (Figure 3).

The letter will include:

1. Statement providing the overall results of the screening (negative, intermediate, or positive).
2. Statement providing further recommended steps on the screening results (timing of next LDCT, or referral)

9. QUALITY REQUIREMENTS AND CONTROL MEASURES FOR LDCT

9.1 Qualifications of Personnel

9.1.1 Qualifications of Study Physicians

Study physicians participating in the trial should meet the following qualifications:

- Must have a valid, active medical license
- A valid GCP licence
- Understanding of the standard practices for lung cancer diagnostics and smoking cessation

9.1.2 Qualifications of Study Nurses

Study nurses participating in the trial should meet the following qualifications:

- Must have a valid, active medical license
- A valid GCP licence
- Understanding of the standard practices for smoking cessation

9.2 CT Equipment Certification and Qualifications

Any imaging equipment or scanner device used to acquire screening images for the study will be certified for use at the time of site qualification.

10. Smoking Cessation Application

10.1 Cessation Application Description and ePRO questionnaire

Participants randomized to the smoking intervention arm will receive a smartphone application for smoking cessation for six months. The smartphone application will also be offered for use after six months of follow-up for the participants randomized to standard-of-care, The cessation application supports the smokers in cessation process and aids them to retain smoking-free lifestyle. The individuals use the app for goal setting, decision-making, information sharing, and empowerment in smoking cessation and, in overall, managing their health.

10.2 Software Development

The developed application is Android and iPhone compatible and administration and database is backed up in a cloud. All of the communications between the mobile app and the supporting backend will be protected by means of an SSL digital certificate on the server, which will provide end-to-end encrypted communications. The application is beta-tested with up to ten users.

10.3 Chacterization of Properties

The app includes 1) an analysis of the individuals smoking history, 2) two questionnaires for lung-related symptom data and psychosocial wellbeing, 3) smoking diary to enchance self-reflection, and 5) virtual supportive channel providing evidence-based data on the benefits of smoking cessation.

The app aims to:

- Provide analytical data for the user's smoking behavior based on the background data provided by the user and smoking behavioral analysis done using the software
- Help out in making a personalized smoking cessation plan based on users smoking behavioral profile
- Provide behavioral support for smoking cessation
- Provide information on the health benefits of smoking cessation

- Provide long-term follow-up data on user’s health status based on symptoms questionnaires and facial photos
- Provide behavioral support for remaining smoking-free after cessation
- Provide data for further development of the application to enhance its performance in smoking cessation and improve the user experience

11. CONCOMITANT INTERVENTIONS

11.1 Prohibited Interventions

- Use of another application for smoking cessation
- Participation in another lung cancer screening pilot or trial

11.2 Trial Subject Withdrawal/Discontinuation

Subject´s participation in the study must be discontinued for any of the following reasons:

- Withdrawal of informed consent
- In the case of any clinical adverse event (AE), laboratory abnormality or illness leading to the conclusion by the investigator that participation into the trial is not the best interest of the subject
- Pregnancy
- Termination of the trial by the Sponsor
- Loss of ability to freely provide informed consent
- Inability to comply with protocol

All trial subjects should comply with the protocol specified follow-up procedures. The only exception to this requirement is a withdrawal of informed consent for all trial procedures or when a subject has lost the ability to consent freely. All data collected until the withdrawal of informed consent will be analyzed and included into trial data.

12. ADVERSE EVENT REPORTING

The objective of adverse event (AE) reporting is the documentation of all events occurring that may compromise the welfare and safety of trial participants. Adverse event reporting is to be distinguished from the collection of data for purposes of analyzing trial endpoints, which is achieved through the recording of specific data elements on case report forms and statistical analyses.

12.1 Definition of Adverse Event

An Adverse Event (AE) is any unfavorable and unintended sign, symptom, or disease temporally associated with the use of a medical treatment or procedure regardless of whether it is considered related to the medical treatment or procedures (attribution of unrelated, possible, propable, or definite).

12.2 Definition of Serious Adverse Event

A Serious Adverse Event (SAE) is any adverse event that results in any of the following:

- Death
- In-patient hospitalization (for reasons other than observations) or prolongation of an existing hospitalization

- A persistent or significant disability or incapacity
- Congenital anomaly/birth defects

12.3 Characterizing Adverse Events by Attribution and Severity

Once identified, the site PI should characterized the AE by attribution (whether it is related to a trial related procedure) and grade of severity. The following guidelines apply:

The attribution of an AE or SAE characterizes its causal relationship to the trial-related procedure as follows:

- Unrelated – clearly NOT related to procedure
- Possible – may be related to procedure
- Probably – likely related to procedure

Grade denotes the severity of the AE and is graded according to the current version of the Common Terminology Criteria for Adverse Events (CTCAE v6.0), or the following cathegories (if the term does NOT appear in the CTCAE v6.0):

1 – Mild
2 – Moderate
3 – Severe
4 – Life-threatening of disabling
5 - Fatal

12.3.1 Direct and Indirect AEs in Screening Imaging Trials

- Complications associated with primary interventions are termed direct AEs
- Screening tests promote downstream, diagnostic interventions; complications associated with these diagnostic interventions are termed indirect AEs
- In this protocol, **only direct adverse events associated with the primary trial interventions will be reported as adverse events**

12.4 Serious Adverse Events

The Investigator must report AEs that meet the definition of an SAE immediately, or within 24 hours of the trial site becoming aware of the SAE.

The AEs defined as serious and which require reporting as an SAE must be reported using a paper SAE Form and emailed to the sponsor (within 24 h of awareness).

The Investigator Site Staff Signature and Task Delegation Log at each trial site will clearly show delegation of responsibilities regarding SAE reporting. A medically qualified person at the trial site identified on the delegation log with this responsibility must assess the SAE. The Principal Investigator or delegated sub-investigators are responsible for the SAE reporting procedures at the site during the trial and must always sign-off on each SAE within five days of reporting even if other site staff have reported the event on behalf of the investigators.

For all SAEs where important or relevant information is missing, active follow-up must be undertaken. The follow-up information of the SAEs must be reported following the same procedure as for the initial reporting. The follow-up report should describe if the SAE has resolved or is continuing, how it was treated, and whether the subject continued bexmarilimab or whether its administration was permanently discontinued.

10.5 AE/SAE Recording and Reporting Period

Details of all AEs will be recorded from the date of consent until the AEs have resolved or no change of the AE status is observed during the last study visit. If the subject is withdrawn from the study, the subject must be followed up for open AEs until the AE has resolved, or no change in subject status is observed over the follow-up period.

13. Device Safety

13.1 Safety Plan

Several measures will be taken to ensure the safety of participants in this study. Participants will undergo safety monitoring during the study, including assessment of the nature, frequency, and severity of adverse device effects. In addition, guidelines for managing adverse device effects, including criteria for the application discontinuation, are provided below.

13.1.1 Risks Associated with the smoking cessation application

The smoking cessation application is fully GDPR compliant.

Potential risks have been assessed according to the sponsor's assessment processes and categorized by severity and probability of occurrence.

Risks related to confidentiality, integrity, and/or availability of the personal data processed by the sponsor cannot be completely eliminated. If you suspect that your personal data has been compromised, please contact the study sponsor.

13.2 Management of Participants Who Experience Adverse Device Events

13.2.1 Safety Parameters and Definitions

Safety assessments will consist of monitoring and recording adverse device effects, including serious adverse device effects.

Certain types of effects require immediate reporting to the Sponsor.

13.2.1.1 Adverse Device Effect

An adverse device effect is any adverse event that is related to the use of an investigational health device (smoking cessation application). This includes adverse events resulting from insufficient or inadequate instructions for use, deployment, implantation, installation, or operation, or any malfunction of the investigational health device. This definition includes any event resulting from use error, or from intentional misuse of the investigational health device.

13.2.1.2 Serious Adverse Device Effects

A serious adverse device effect is an adverse device effect that has resulted in any of the consequences characteristic of a serious adverse event. Serious adverse device effects are either anticipated or unanticipated.

An anticipated serious adverse device effect is a serious adverse device effect, which by its nature, incidence, severity, or outcome has been identified. An unanticipated serious adverse device effect is a serious adverse device effect, which by its nature, incidence, severity, or outcome, has not been identified in the Risk Assessment Report. This includes unanticipated procedure-related serious adverse events; that is, serious adverse events occurring during the study procedure that are unrelated to any malfunction or misuse of the investigational medical device.

13.2.1.3 Anticipated Adverse Device Effects and Anticipated Serious Adverse Device Effects
Per the investigational device’s labelling, no serious adverse device effects are anticipated.

13.2.1.4 Device Deficiency
A device deficiency is defined as any inadequacy in the identity, quality, durability, reliability, usability, safety, or performance of an investigational device, including malfunction, use errors, or inadequacy of manufacturer-supplied information.

All device deficiencies must be recorded on the eCRF. An assessment must be made as to whether the device deficiency could have led to a serious adverse device effect if:

- Appropriate action had not been taken,
- Intervention had not occurred, or,
- Circumstances had been less fortunate.

13.2.1.5 Serious Health Threat
A serious health threat is defined as a signal from any adverse device effect or device deficiency that indicates an imminent risk of death or a serious deterioration in the health in participants, users or other persons, and that requires prompt remedial action for other participants, users or other persons. This would include events that are of significant and unexpected nature such that they become alarming as a potential serious health hazard or possibility of multiple deaths occurring at short intervals.

13.2.2 Follow-up of Participants after Adverse Device Effects

13.2.2.1 Investigator Follow-up
The investigator should follow each adverse device effect until the event has resolved to baseline or better, the event is assessed as stable by the investigator, the participant is lost to follow-up, or the participant withdraws consent. Every effort should be made to follow all serious adverse device effects considered to be related to the device or study-related procedures until a final outcome can be reported.

During the event reporting period, resolution of adverse device effects (with dates) should be documented on the CRF and in the participant's medical record to facilitate source data verification.

13.2.2.2 Sponsor Follow-up
For serious adverse device effects and device deficiencies that could have led to a serious adverse device effect, the Sponsor or a designee may follow up by telephone, fax, email, and/or a monitoring visit to obtain additional case details and outcome information (e.g., from hospital discharge summaries, consultant reports, autopsy reports) in order to perform an independent medical assessment of the reported case.

13.2.3 Adverse Device Effects That Occur after the Adverse Device Effect Reporting Period
The Sponsor should be notified if the investigator becomes aware of any serious adverse device effect that occurs after the end of the adverse device effect reporting period (withdrawal from the study), and if the event is believed to be related to the device. These events should be reported through use of the CRF.

14. DATA HANDLING AND RECORD KEEPING

14.1 Data Collection
All relevant data related to safety and efficacy including laboratory values and clinical parameters should be recorded in the medical records and be captured in the CRF. The site is responsible for completing

the CRF forms in a timely manner. All data on the eCRFs must be verifiable in the source data/hospital or patient records, unless eCRF data are declared as source data.

14.2 Device Data

Data will be collected within a cloud-based platform(s), for processing, analysis and storage, managed by the sponsor. Only identified and trained users access this system. Device data may be analyzed on an ongoing basis for the purpose of device changes.

14.3 Archiving

It is the responsibility of the Principal Investigator to ensure all essential trial documentation and source records (e.g. signed Patient Information and Consent Documents, Investigator Site Files, patients' hospital notes, copies of CRFs etc.) are stored in secure archives for 15 years after the end of the trial. However, these documents should be retained for a longer period if required by applicable legal requirements. The Sponsor's approval is required prior to transfer or destruction of the documents.

15. QUALITY MANAGEMENT

This trial is to be conducted according to the ICH harmonized tripartite guideline for good clinical practice E6(R2) and the European Union directive 2001/20/EC to ensure that the rights, safety and well-being of trial subjects are protected, consistent with the principles that have their origin in the Declaration of Helsinki, and that the clinical trial data are credible.

For the trial to start, all required documentation must be approved by the relevant ethical committees and competent authorities, in accordance with local legal requirements. The Sponsor must ensure that all ethical and legal requirements have been met before the first subject is enrolled in the trial.

This protocol is to be followed exactly. To alter the protocol, amendments must be written, receive approval from the appropriate personnel, and receive ethical committee/competent authority approval prior to implementation (if appropriate).

Administrative changes (not affecting the subject benefit/risk ratio) may be made without the need for a formal amendment. All amendments will be distributed to all protocol recipients, with appropriate instructions.

15.1 Site Set-up and Initiation

All participating Investigators will be asked to sign the necessary agreements and supply a current CV to the Sponsor or representative of the Sponsor. All members of the site research team that will perform study specific activities will also be required to sign the "Site Staff Signature and Task Delegation Log". Prior to commencing recruitment, all sites will undergo a process of initiation. Key members of the site research team will be required to be trained by the Sponsor, or a representative of the Sponsor, covering aspects of the trial design, protocol procedures, Adverse Event reporting, collection and reporting of data and record keeping. The investigators are responsible for ensuring that appropriate training relevant to the study is given to the medical, nursing and other personnel involved in the study. The investigators will also ensure that any information relevant to the conduct of the study is forwarded to the sub-investigators and other relevant study center personnel. Sites will be provided with an Investigator Site File containing essential documentation, instructions, and other documentation required for the conduct of the trial. The Sponsor or representative of the Sponsor must be informed immediately of any change in the site research team.

15.2 On-Site Monitoring

A separate Site Monitoring and Management Plan is available where full details of the monitoring activities are described. The trial is conducted according to the GCP and quality standards. Monitors must ensure that the required documentation and trial site training has been done before the site can start enrolling subjects. During the treatment phase, the site(s) will be monitored to ensure protocol adherence and to verify the source data. Any issues in compliance will be reported to the Sponsor.

By participating into this trial, the Investigator(s) and the site(s) conducting this trial will permit trial-related monitoring, audits, Institutional Review Board or equivalent review, and regulatory inspection(s) and provide direct access to source data/documents. A reasonable time must be given for the site(s) to prepare for such activities.

15.3 Audit and Inspection

The Investigator will permit trial-related monitoring, audits, ethical review, and regulatory inspection(s) at their site, providing direct access to source data/documents. Sites are also requested to notify the Sponsor of any relevant regulatory authority inspections.

15.4 Notification of Serious Breaches

The sites and the Sponsor of the trial will report any serious breaches or protocol/ICHGCP deviations in accordance with the local laws and current regulations. A "serious breach" is a breach which is likely to effect to a significant degree:

- The safety and rights of a subject.
- The reliability and robustness of the data generated in the clinical trial.

Where the Sponsor is investigating whether or not a serious breach has occurred, sites are also requested to cooperate with the Sponsor in providing sufficient information to report the breach to the relevant regulatory authorities/ethics committees where required and in undertaking any corrective and/or preventive action.

16. END OF TRIAL DEFINITION

The end of trial will be the date the overall survival and lung cancer data of the last subject has been collected. The Sponsor will notify relevant regulatory authorities and ethics committees that the trial has ended at the appropriate time and will provide them with a summary of the clinical trial report within 12 months of the end of trial.

The Sponsor reserves the right to stop the trial at any time on the basis of new information regarding safety or efficacy (e.g., discovery of an unexpected, significant or unacceptable risk to the subjects enrolled in the trial), or if trial progress is unsatisfactory (e.g., failure to enroll subjects at an acceptable rate), or for other valid reasons (e.g., Sponsor decides to suspend or discontinue development of the drug). After such a decision is made, the Investigator must inform all on-trial subjects within one (1) week. All delivered trial materials must be collected and all e-CRF pages completed to the extent possible.

17. STATISTICAL CONSIDERATIONS

17.1 Trial Population

The trial subject population includes patients who have marked smoking history and are current smokers and who adhere to the trial inclusion and exclusion criteria.

For the purpose of analyses the following populations are defined:

Efficacy evaluable population – Includes all randomized and undergone at least one round LDCT screening.

17.2 Analysis of Outcome Measures

17.2.1 Primary Outcomes

- The percentage of subjects who are actively smoking at 3 months after inclusion between the study arms
- The percentage of subjects who are actively smoking at 6 months after inclusion between the study arms

Eligible subjects are randomized (1:1) with stratification according to pack years (<30py or ≥30py), and age (<65 or ≥65v) to smartphone based smoking cessation and control arm.

The study is powered (80%) with 155 subjects to detect 15% difference in (75 vs. 90%) in the number of active smokers at three and six months after inclusion with 90% confidence. With the expected dropout rate, the sample size is adjusted to 200.

17.2.2 Secondary Outcomes

- The percentage of subjects who have reduced the amount of smoked cigarettes/d at 3 and 6 months after inclusion between the study arms
- The magnitude and percentage of reduction in cigrattes consumed/d at 3 and 6 months after inclusion between the study
- Sensitivity and positive predictive value of CT-screening in the whole cohort
- Costs related to CT screening including additional investigations

17.2.3 Exploratory Outcomes

- Specificity of CT-screening in the whole cohort
- Lung cancer incidence (stage specific) and survival
- Number of additional CT-scans, PET-CT scans, bronchoscopies, needle biopsies, and their results (positive vs. negative for cancer) initiated based on screening CT-findings
- Percentage of subjects identified through newspaper advertisement and referral from primary care physician or occupational health physician
- Quality of life (QoL)
- Spectrum and grading of ePRO symptoms
- Correlation of ePRO symptoms to CT screen results
- Biomarker analysis of blood such as ctDNA
- AI-based evaluation of LDCT scans

17.3 Analysis of Efficacy Parameters

The analyzes for primary end-points will be carried out when the last study subject has reached the milestone of analyzes and all the CRF collected information related to end-point is available.

The analyzes for secondary and explotory outcomes will be carried out after the last subject has reached the second screening round visit and/or end-of-study (3y), which ever is feasible, and all the CRF collected information related to end-point is available.

17.4 Planned Interim Analysis

There is no predefined interim analysis. Interim analyses on efficacy, and selected biomarkers may be provided on ongoing basis prior to completion of the trial in order to expedite conclusions and to support trial presentations or publications.

18. ACCESS TO SOURCE DATA AND RELATED DOCUMENTS

By participating into this trial, the Investigator(s) and the site(s) conducting this trial will permit trial-related monitoring, audits, Institutional Review Board or equivalent review, and regulatory inspection(s) and provide direct access to source data/documents. A reasonable time must be given for the site(s) to prepare for such activities.

19. TRIAL ORGANIZATIONAL STRUCTURE

19.1 Sponsor

The Sponsor of the trial is Oulu University Hospital. The Sponsor may delegate duties to trial sites.

19.2 Finance

The Sponsor will make contracts with trial sites.

20. ETHICAL CONSIDERATION

The trial will be performed in accordance with the recommendations guiding physicians in biomedical research involving human subjects, adopted by the 18th World Medical Association General Assembly, Helsinki, Finland, June 1964, amended at the 64th World Medical Association General Assembly, Fortaleza, Brazil, October 2013 (website: https://www.wma.net/policies-post/wma-declaration-of-helsinki-ethical-principles-formedical-research-involving-human-subjects/).

The trial will be conducted in accordance with the European Union directive 2001/20/EC, data protection regulation (Regulation (EU) 2016/679) local laws, and the ICH GCP E6(R2). This trial will be carried out under a Clinical Trial Authorization in accordance with local regulations. The protocol will be submitted to and approved by the corresponding ethical committees.
Before any subjects are enrolled into the trial, the Principal Investigator at each site is required to obtain corresponding approval. Sites will not be permitted to enroll subjects until written confirmation of approval is received.

It is the responsibility of the Principal Investigator to ensure that all subsequent amendments gain the necessary local approval. This does not affect the individual clinicians' responsibility to take immediate action if thought necessary to protect the health and interest of individual subjects.

21. CONFIDENTIALITY AND DATA PROTECTION

All trial findings and documents will be regarded as confidential. The Investigator and members of their research team must not disclose any information without prior written approval from the Sponsor. The Investigator must maintain documents not for submission to the Sponsor in strict confidence. In the case of specific issues and/or queries from the regulatory authorities, it will be necessary to have access to the complete trial records, provided that subject confidentiality is protected.

Subjects will be identified on the CRF and other documents submitted to the Sponsor or Sponsor's representative by their trial subject number. Documents that identify the subject must not to be submitted to the Sponsor and must be maintained in confidence by the Investigator. Only the Investigator and authorized staff will be able to enter and correct data in the CRF.

CRF documents should be completed for each subject included in the trial and should reflect the latest observations on the subjects participating in the trial. Therefore, the CRF is to be completed as soon as possible during or immediately after the subject's visit or assessment. The Investigator must verify that all data entries in the CRF are accurate and correct. If some assessments cannot be done, or if certain information is unavailable, not applicable or unknown, the Investigator should indicate this in the CRF.

All discrepancies must be resolved online directly by the Investigator or by staff authorized to do this by Delegation of Authority. The Investigator will be required to sign off the clinical data recorded in the CRF.

22. PUBLICATION POLICY

Any manuscript, abstract or other publication or presentation of results or information arising in connection with the trial (including any ancillary trial involving trial subjects) must be prepared in conjunction with the trial Sponsor. The Sponsor will review the communications for accuracy to avoid potential discrepancies with submissions to health authorities, verify that confidential information is not accidentally disclosed, and provide any relevant supplementary information. The Sponsor's comments on the proposed publication shall be considered in good faith by the authors. The Sponsor may delay such submission by a maximum of 90 days if it reasonably believes that publication of results may compromise its intellectual property rights or may insist that such information or data is removed from the proposed publication. Publication of the results will not include confidential information without the permission of the Sponsor.

The original CRF pages and all data generated during the trial under this protocol will become the property of the Sponsor.

The Sponsor may announce quality-assured summary data in order to comply with the requirements of financial regulatory authorities, while ensuring so far as possible that such announcements will not compromise the Investigators' ability to publish the data in appropriate scientific forums. Authorship of

communications arising from the trial-related data and subsequent analysis may include members from the contributing site(s) including basic research laboratories and Sponsor's personnel.

20. REFERENCE

1. National Lung Cancer Screening Trial Research.D.R. Aberle, A.M. Adams, C.D. Berg, W.C. Black, J.D. Clapp, R.M. Fagerstrom, I.F. Gareen, C. Gatsonis, P.M. Marcus, J.D. Sicks, Reduced lung-cancer mortality with low-dose computed tomographic screening. N. Engl. J. Med. 365(2011) 395-409
2. A.C. Villanti, Y. Jiang, D.B. Abrams, B.S: Pyenson, A cost-utility analysis of lung cancer screening and the additional benefits of incorporating smoking cessation interventions. PLoS One 8(2013)e71379
3. S.R. Land, P.M. Marcus, Cancer screening and diagnosis: opportunities for smoking cessation intervention. J. Clin. Oncol. 33(2015)1631-1632
4. Naslund JA, Kim SJ, Ascbrenner KA, McCulloch LJ, Brunette MF, Dallery J, Bartels SJ, Marsch L. Systematic review of social media interventions for smoking cessation. Addictive Behaviors 73(2017) 81-93
5. de Koning H.J., van der Aalst C.M., de Jong P.A., Scholten E.T., Nackaerts K., Heuvelmans M.A., et al. Reduced Lung-Cancer Mortality with Volume CT Screening in a Randomized Trial N Engl J Med 2020; 382:503-513
6. Lancaster T, Stead LF. Individual behavioural counselling for smoking cessation (Cochrane Review 2008).

APPENDIX 1 - WMA DECLARATION OF HELSINKI

Adopted by the 18th WMA General Assembly, Helsinki, Finland, June 1964 and amended by the:
29th WMA General Assembly, Tokyo, Japan, October 1975
35th WMA General Assembly, Venice, Italy, October 1983
41st WMA General Assembly, Hong Kong, September 1989
48th WMA General Assembly, Somerset West, Republic of South Africa, October 1996
52nd WMA General Assembly, Edinburgh, Scotland, October 2000
53rd WMA General Assembly, Washington DC, USA, October 2002 (Note of Clarification added)
55th WMA General Assembly, Tokyo, Japan, October 2004 (Note of Clarification added)
59th WMA General Assembly, Seoul, Republic of Korea, October 2008
64th WMA General Assembly, Fortaleza, Brazil, October 2013

Preamble

1. The World Medical Association (WMA) has developed the Declaration of Helsinki as a statement of ethical principles for medical research involving human subjects, including research on identifiable human material and data. The Declaration is intended to be read as a whole and each of its constituent paragraphs should be applied with consideration of all other relevant paragraphs.

2. Consistent with the mandate of the WMA, the Declaration is addressed primarily to physicians. The WMA encourages others who are involved in medical research involving human subjects to adopt these principles.

General Principles

3. The Declaration of Geneva of the WMA binds the physician with the words, “The health of my patient will be my first consideration,” and the International Code of Medical Ethics declares that, “A physician shall act in the patient’s best interest when providing medical care.”

4. It is the duty of the physician to promote and safeguard the health, well-being and rights of patients, including those who are involved in medical research. The physician’s knowledge and conscience are dedicated to the fulfilment of this duty.

5. Medical progress is based on research that ultimately must include studies involving human subjects.

6. The primary purpose of medical research involving human subjects is to understand the causes, development and effects of diseases, and improve preventive, diagnostic and therapeutic interventions (methods, procedures and treatments). Even the best proven interventions must be evaluated continually through research for their safety, effectiveness, efficiency, accessibility and quality.

7. Medical research is subject to ethical standards that promote and ensure respect for all human subjects and protect their health and rights.

8. While the primary purpose of medical research is to generate new knowledge, this goal can never take precedence over the rights and interests of individual research subjects.

9. It is the duty of physicians who are involved in medical research to protect the life, health, dignity, integrity right to self-determination, privacy, and confidentiality of personal information of research subjects. The responsibility for the protection of research subjects must always rest with the physician or other health care professionals and never with the research subjects, even though they have given consent.

10. Physicians must consider the ethical, legal and regulatory norms and standards for research involving human subjects in their own countries as well as applicable international norms and standards. No national or international ethical, legal or regulatory requirement should reduce or eliminate any of the protections for research subjects set forth in this Declaration.

11. Medical research should be conducted in a manner that minimizes possible harm to the environment.
12. Medical research involving human subjects must be conducted only by individuals with the appropriate ethics and scientific education, training and qualifications. Research on patients or healthy volunteers requires the supervision of a competent and appropriately qualified physician or other health care professional.
13. Groups that are underrepresented in medical research should be provided appropriate access to participation in research.
14. Physicians who combine medical research with medical care should involve their patients in research only to the extent that this is justified by its potential preventive, diagnostic or therapeutic value and if the physician has good reason to believe that participation in the research study will not adversely affect the health of the patients who serve as research subjects.
15. Appropriate compensation and treatment for subjects who are harmed as a result of participating in research must be ensured.

Risks, Burdens and Benefits
16. In medical practice and in medical research, most interventions involve risks and burdens. Medical research involving human subjects may only be conducted if the importance of the objective outweighs the risks and burdens to the research subjects.
17. All medical research involving human subjects must be preceded by careful assessment of predictable risks and burdens to the individuals and groups involved in the research in comparison with foreseeable benefits to them and to other individuals or groups affected by the condition under investigation. Measures to minimize the risks must be implemented. The risks must be continuously monitored, assessed and documented by the researcher.
18. Physicians may not be involved in a research study involving human subjects unless they are confident that the risks have been adequately assessed and can be satisfactorily managed. When the risks are found to outweigh the potential benefits or when there is conclusive proof of definitive outcomes, physicians must assess whether to continue, modify or immediately stop the study.

Vulnerable Groups and Individuals
19. Some groups and individuals are particularly vulnerable and may have an increased likelihood of being wronged or of incurring additional harm. All vulnerable groups and individuals should receive specifically considered protection.
20. Medical research with a vulnerable group is only justified if the research is responsive to the health needs or priorities of this group and the research cannot be carried out in a non-vulnerable group. In addition, this group should stand to benefit from the knowledge, practices or interventions that result from the research.

Scientific Requirements and Research Protocols
21. Medical research involving human subjects must conform to generally accepted scientific principles, be based on a thorough knowledge of the scientific literature, other relevant sources of information, and adequate laboratory and, as appropriate, animal experimentation. The welfare of animals used for research must be respected.
22. The design and performance of each research study involving human subjects must be clearly described and justified in a research protocol. The protocol should contain a statement of the ethical considerations involved and should indicate how the principles in this Declaration have been addressed. The protocol should include information regarding funding, sponsors, institutional affiliations, potential

conflicts of interest, incentives for subjects and information regarding provisions for treating and/or compensating subjects who are harmed as a consequence of participation in the research study.
In clinical trials, the protocol must also describe appropriate arrangements for post-trial provisions.

Research Ethics Committees

23. The research protocol must be submitted for consideration, comment, guidance and approval to the concerned research ethics committee before the study begins. This committee must be transparent in its functioning, must be independent of the researcher, the sponsor and any other undue influence and must be duly qualified. It must take into consideration the laws and regulations of the country or countries in which the research is to be performed as well as applicable international norms and standards, but these must not be allowed to reduce or eliminate any of the protections for research subjects set forth in this Declaration.
The committee must have the right to monitor ongoing studies. The researcher must provide monitoring information to the committee, especially information about any serious adverse events. No amendment to the protocol may be made without consideration and approval by the committee. After the end of the study, the researchers must submit a final report to the committee containing a summary of the study's findings and conclusions.

Privacy and Confidentiality

24. Every precaution must be taken to protect the privacy of research subjects and the confidentiality of their personal information.

Informed Consent

25. Participation by individuals capable of giving informed consent as subjects in medical research must be voluntary. Although it may be appropriate to consult family members or community leaders, no individual capable of giving informed consent may be enrolled in a research study unless he or she freely agrees.

26. In medical research involving human subjects capable of giving informed consent, each potential subject must be adequately informed of the aims, methods, sources of funding, any possible conflicts of interest, institutional affiliations of the researcher, the anticipated benefits and potential risks of the study and the discomfort it may entail, post-study provisions and any other relevant aspects of the study. The potential subject must be informed of the right to refuse to participate in the study or to withdraw consent to participate at any time without reprisal. Special attention should be given to the specific information needs of individual potential subjects as well as to the methods used to deliver the information.
After ensuring that the potential subject has understood the information, the physician or another appropriately qualified individual must then seek the potential subject's freely given informed consent, preferably in writing. If the consent cannot be expressed in writing, the non-written consent must be formally documented and witnessed. All medical research subjects should be given the option of being informed about the general outcome and results of the study.

27. When seeking informed consent for participation in a research study the physician must be particularly cautious if the potential subject is in a dependent relationship with the physician or may consent under duress. In such situations the informed consent must be sought by an appropriately qualified individual who is completely independent of this relationship.

28. For a potential research subject who is incapable of giving informed consent, the physician must seek informed consent from the legally authorized representative. These individuals must not be included in a research study that has no likelihood of benefit for them unless it is intended to promote the health of the group represented by the potential subject, the research cannot instead be performed

with persons capable of providing informed consent, and the research entails only minimal risk and minimal burden.

29. When a potential research subject who is deemed incapable of giving informed consent is able to give assent to decisions about participation in research, the physician must seek that assent in addition to the consent of the legally authorized representative. The potential subject's dissent should be respected.

30. Research involving subjects who are physically or mentally incapable of giving consent, for example, unconscious patients may be done only if the physical or mental condition that prevents giving informed consent is a necessary characteristic of the research group. In such circumstances the physician must seek informed consent from the legally authorized representative. If no such representative is available and if the research cannot be delayed, the study may proceed without informed consent provided that the specific reasons for involving subjects with a condition that renders them unable to give informed consent have been stated in the research protocol and the study has been approved by a research ethics committee. Consent to remain in the research must be obtained as soon as possible from the subject or a legally authorized representative.

31. The physician must fully inform the patient which aspects of their care are related to the research. The refusal of a patient to participate in a study or the patient's decision to withdraw from the study must never adversely affect the patient-physician relationship.

32. For medical research using identifiable human material or data, such as research on material or data contained in biobanks or similar repositories, physicians must seek informed consent for its collection, storage and/or reuse. There may be exceptional situations where consent would be impossible or impracticable to obtain for such research. In such situations the research may be done only after consideration and approval of a research ethics committee.

Use of Placebo

33. The benefits, risks, burdens and effectiveness of a new intervention must be tested against those of the best proven intervention(s), except in the following circumstances:

Where no proven intervention exists, the use of placebo, or no intervention, is acceptable; or

Where for compelling and scientifically sound methodological reasons the use of any intervention less effective than the best proven one, the use of placebo, or no intervention is necessary to determine the efficacy or safety of an intervention and the patients who receive any intervention less effective than the best proven one, placebo, or no intervention will not be subject to additional risks of serious or irreversible harm as a result of not receiving the best proven intervention. Extreme care must be taken to avoid abuse of this option.

Post-Trial Provisions

34. In advance of a clinical trial, sponsors, researchers and host country governments should make provisions for post-trial access for all participants who still need an intervention identified as beneficial in the trial. This information must also be disclosed to participants during the informed consent process.

Research Registration and Publication and Dissemination of Results

35. Every research study involving human subjects must be registered in a publicly accessible database before recruitment of the first subject.

36. Researchers, authors, sponsors, editors and publishers all have ethical obligations with regard to the publication and dissemination of the results of research. Researchers have a duty to make publicly available the results of their research on human subjects and are accountable for the completeness and accuracy of their reports. All parties should adhere to accepted guidelines for ethical reporting. Negative and inconclusive as well as positive results must be published or otherwise made publicly available.

Sources of funding, institutional affiliations and conflicts of interest must be declared in the publication. Reports of research not in accordance with the principles of this Declaration should not be accepted for publication.

Unproven Interventions in Clinical Practice

37. In the treatment of an individual patient, where proven interventions do not exist or other known interventions have been ineffective, the physician, after seeking expert advice, with informed consent from the patient or a legally authorized representative, may use an unproven intervention if in the physician's judgement it offers hope of saving life, re-establishing health or alleviating suffering. This intervention should subsequently be made the object of research, designed to evaluate its safety and efficacy. In all cases, new information must be recorded and, where appropriate, made publicly available.

https://www.wma.net/policies-post/wma-declaration-of-helsinki-ethical-principles-for-medical-research-involving-human-subjects/

APPENDIX 2 - DEFINITION OF ADVERSE EVENTS

Adverse Event (AE)

Any untoward medical occurrence in a patient or clinical trial subject administered a medicinal product and which does not necessarily have a causal relationship with this treatment. An AE can therefore be any unfavorable and unintended sign (including abnormal laboratory findings), symptom or disease temporally associated with the use of an investigational medicinal product, whether or not related to the investigational medicinal product.

Serious Adverse Event (SAE)

Any untoward medical occurrence or effect that at any dose:

- Results in death
- Is life-threatening*
- Requires hospitalization** or prolongation of existing inpatients' hospitalization
- Results in persistent or significant disability or incapacity
- Is a congenital anomaly/birth defect
- Or is otherwise considered medically significant by the Investigator***

Comments:

The term severe is often used to describe the intensity (severity) of a specific event. This is not the same as serious, which is based on patients/event outcome or action criteria.

* Life threatening in the definition of an SAE refers to an event in which the patient was at risk of death at the time of the event; it does not refer to an event that hypothetically might have caused death if it were more severe.

**Hospitalization is defined as an unplanned, formal inpatient admission, even if the hospitalization is a precautionary measure for continued observation. Thus, hospitalization for protocol treatment (e.g. line insertion), elective procedures (unless brought forward because of worsening symptoms) or for social reasons (e.g. respite care) are not regarded as an SAE.

*** Medical judgment should be exercised in deciding whether an AE is serious in other situations. Important AEs that are not immediately life threatening or do not result in death or hospitalization but may jeopardize the subject or may require intervention to prevent one of the other outcomes listed in the definition above, should be considered serious.

APPENDIX 3 - COMMON TOXICITY CRITERIA GRADINGS

Toxicities will be recorded according to the NCI-CTCAE version 6.0. The full CTCAE document is available on the National Cancer Institute (NCI) website, the following address was correct when this version of the protocol was approved: http://ctep.cancer.gov/protocolDevelopment/electronic_application
