# Supplementary material for: Effect of Lung Cancer Screening, Smoking Cessation, and Cessation Smartphone App to Health-Related Quality of Life Among Heavy Smokers: Randomized Controlled Trial
Source: J Med Internet Res. 2026 Jan 20;28:e81687. doi: 10.2196/81687 (PMC12818497; doi:10.2196/81687)
Supplement: Multimedia Appendix 3 [file jmir-v28-e81687-s003.docx]

**Table S1.** Demographics of the LDCT-SC-FI trial were participants randomized to a yearly low-dose CT with standard smoking cessation (Control) or the same LDCT screening approach combined to a smartphone application-based smoking cessation (Application).

|  | **Application** | **Control** |
| --- | --- | --- |
|  | n (%)/mean | n (%)/mean |
| All | 101 | 99 |
| Age | 61.35 | 59.96 |
| Gender |  |  |
| Male | 48 (47.5) | 50 (50.5) |
| Female | 53 (52.5) | 49 (49.5) |
| Relationship status |  |  |
| Single | 32 (31.7) | 26 (26.3) |
| In a relationship | 69 (68.3) | 73 (73.7) |
| ICT skills |  |  |
| Novice | 7 (6.9) | 8 (8.1) |
| Average | 51 (50.5) | 47 (47.5) |
| Exprienced | 43 (42.6) | 44 (44.4) |
| How many cigarettes/d | 15.53 | 16.70 |
| Pack years | 32.72 | 33.57 |
| Fagerstöm test | 2.75 | 2.92 |
| How many times quitted | 2.96 | 3.44 |
| Smoking status at one year | 93 (100) | 93 (100) |
| Smokers | 76 (81.7) | 82 (87.2) |
| Non-smokers | 17 (18.3) | 12 (12.8) |

**Table S2.** Reliability analysis of HRQoL data of QLQ-C30+LC13 and EQ-5D of the LDCT-SC-FI study population.

| **PROM measure** | **Number of items** | **Scoring** | **Interpretation** | **Cronbach’s alpha** |
| --- | --- | --- | --- | --- |
| **QLQ-C30** |  |  |  |  |
| Physical functioning | 5 items (1-5) | 1-4 | Higher=better functioning | 0.877 |
| Role functioning | 2 items (6-7) | 1-4 | ” | 0.791 |
| Emotional functioning | 4 items (21-24) | 1-4 | ” | 0.861 |
| Cognitive functioning | 2 items (20,25) | 1-4 | ” | 0.776 |
| Social functioning | 2 items (26,27) | 1-4 | ” | 0.772 |
| Fatigue | 3 items (10,12,18) | 1-4 | Higher=more symptoms | 0.853 |
| Nausea and vomiting | 2 items (14,15) | 1-4 | ” | 0.415 |
| Pain | 2 items (9,19) | 1-4 | ” | 0.864 |
| Dyspnea | 1 item (8) | 1-4 | ” | 0.695 |
| Insomnia | 1 item (11) | 1-4 | ” | 0.721 |
| Appetite loss | 1 item (13) | 1-4 | ” | **0.432** |
| Constipation | 1 item (16) | 1-4 | ” | 0.789 |
| Diarrhea | 1 item (17) | 1-4 | ” | 0.645 |
| Financial difficulties | 1 item (28) | 1-4 | ” | 0.756 |
| Global health status/QoL | 2 items (29,30) | 1-7 | Higher=better QoL | 0.826 |
| **LC13** |  |  |  |  |
| Coughing | 1 item (31) | 1-4 | Higher=worse symptoms | 0.620 |
| Hemoptysis (Coughing blood) | 1 item (32) | 1-4 | ” | **1,91*10^−14^** |
| Dyspnea (shortness of breath): | 3 item (33–35) | 1-4 | ” | 0.791 |
| Sore mouth | 1 item (36) | 1-4 | ” | **0.479** |
| Dysphagia (difficulty swallowing) | 1 item (37) | 1-4 | ” | 0.643 |
| Peripheral neuropathy (numbness) | 1 item (38) | 1-4 | ” | 0.733 |
| Alopecia (hair loss) | 1 item (39) | 1-4 | ” | 0.543 |
| Pain in chest | 1 item (40) | 1-4 | ” | 0.620 |
| Pain in arm/shoulder | 1 item (41) | 1-4 | ” | 0.678 |
| Pain in other parts | 1 item (42) | 1-4 | ” | 0.655 |
| **EQ-5D 3L** |  |  |  |  |
| Mobility | 1 item (1) | 1-3 | 1=no;3=worst problems | 0.639 |
| Self-care | 1 item (2) | 1-3 | ” | 0.650 |
| Usual activities | 1 item (3) | 1-3 | ” | 0.750 |
| Pain/discomfort | 1 item (4) | 1-3 | ” | 0.704 |
| Anxiety/depression | 1 item (5) | 1-3 | ” | 0.706 |
| EQ-5D index score | 5 items | 0-1 | ” | 0.650 |
| EQ-5D Finland | 5 items | 0-1 | Higher=better QoL | 0.779 |

**Table S3.** Correlation between EQ-5D index score change and QLQ-C30 change at 1y. A 10-point change in scores of QLQ-C30 is considered meaningful, thus, a 10% cut-off was selected while a 5% change in EQ-5D index score is considered significant in Finland.

|  | |  | **QLQ-C30 GHS** (10% change) |  | ***P*-value*** | **Effect size^#^** |
| --- | --- | --- | --- | --- | --- | --- |
|  | | Improved  n (%) | No change  n (%) | Declined  n (%) |  |  |
| **EQ-5D index score** (5% change) | |  |  |  |  |  |
| Improved | | 15 (32.6) | 25 (54.3) | 6 (13.0) | **.001** | 0.071(.01-.15) |
| No change | 14 (15.6) | 65 (72.2) | 11 (12.2) |  |  |  |
| Declined | 4 (8.0) | 30 (60.0) | 16 (32.0) |  |  |  |

*Pearson Chi-Square test; ^#^Partial Eta-squared with ANOVA

**Table S4.** Changes in Quality of Life based on QLQ-C30 GHS and EQ-5D index score between baseline and at 1y according to randomization arm excluding individuals from the control arm with reported use of the smoking cessation application. A 10-point change in scores of QLQ-C30 is considered meaningful, thus, a 10% cut-off was selected while a 5% change in EQ-5D index score is considered significant in Finland.

|  | **Control**  mean/n (SD/%) | **Application**  mean/n (SD/%) | ***P*-value*** | **Effect size^#^** (95% CI) | **Adjusted *P-*value^$^** | **Adjusted Effect size^$^** |
| --- | --- | --- | --- | --- | --- | --- |
| QLQ-C30 GHS |  |  |  |  |  |  |
| Baseline | 70.69 (15.69) | 74.17 (16.31) | .140 | 0.012(.00-.06) |  |  |
| 1y | 68.14 (20.58) | 74.82 (19.23) | **.029** | 0.028(.00-.09) | .143 | 0.013 |
| QLQ-C30 GHS (10% change) | 79 (100) | 93 (100) |  |  |  |  |
| Improved | 7 (8.9) | 22 (23.7) | **.036** | 0.023(.00-.08) |  |  |
| No change | 58 (72.2) | 56 (60.2) |  |  |  |  |
| Declined | 15 (19.0) | 15 (16.1) |  |  |  |  |
| EQ-5D index score |  |  |  |  |  |  |
| Baseline | 0.751 (0.198) | 0.759 (0.206) | .802 | 0.00(.00-.02) |  |  |
| 1y | 0.729 (0.202) | 0.799 (0.197) | **.022** | 0.030(.00-.10) | **.014** | 0.035 |
| EQ-5D index score (5% change) | 79 (100) | 93 (100) |  |  |  |  |
| Improved | 14 (17.7) | 29 (31.2) | .087 | 0.027(.00-.09) |  |  |
| No change | 41 (51.9) | 45 (48.4) |  |  |  |  |
| Declined | 24 (30.4) | 19 (20.4) |  |  |  |  |

*ANOVA/Pearson Chi-Square test; ^#^Partial Eta-squared; ^$^ANCOVA adjusted for baseline QLQ-C30 or EQ-5D scores
